# Cluster Size of Amylopectin and Nanosized Amylopectin Fragments Characterized by Pyrene Excimer Formation

**DOI:** 10.3390/polym14163418

**Published:** 2022-08-21

**Authors:** Damin Kim, Jean Duhamel

**Affiliations:** Department of Chemistry, Institute for Polymer Research, Waterloo Institute of Nanotechnology, University of Waterloo, Waterloo, ON N2L 3G1, Canada

**Keywords:** pyrene excimer fluorescence, amylopectin, clusters of helices, building blocks

## Abstract

Amylopectin from waxy corn and the three nanosized amylopectin fragments (NAFs)—NAF(56), NAF(20), and NAF(8)—from waxy corn starch with a hydrodynamic diameter of 227, 56, 20, and 8 nm, respectively, were randomly labeled with 1-pyrenebutyric acid. The efficiency of these pyrene-labeled amylopectin-based polysaccharides (Py-A*b*PS) for pyrene excimer formation (PEF) upon diffusive encounter between an excited and a ground-state pyrene increased with increasing concentration of unlabeled NAF(56) in Py-A*b*PS dispersions in DMSO. Fluorescence decay analysis of the Py-A*b*PS dispersions in DMSO prepared with increasing [NAF(56)] yielded the maximum number (*N*_blob_^exp^) of anhydroglucose units (AGUs) separating two pyrene-labeled AGUs while still allowing PEF. Comparison of *N*_blob_^exp^ with *N*_blob_^theo^, obtained by conducting molecular mechanics optimizations on helical oligosaccharide constructs with HyperChem, led to a relationship between the interhelical distance (*d*_h-h_) in a cluster of oligosaccharide helices, [NAF(56)], and the number of helices in a cluster. It was found that the A*b*PSs were composed of building blocks made of 3.5 (±0.9) helices that self-assembled into increasingly larger clusters with increasing [NAF(56)]. The ability of PEF-based experiments to yield the cluster size of A*b*PSs provides a new experimental means to probe the interior of A*b*PSs at the molecular level.

## 1. Introduction

Amylopectin is the second most abundant polysaccharide on earth after cellulose [1]. It is an abundant, biodegradable, renewable, and safe material that is increasingly viewed as a potential feedstock by the plastic industry to be used in several applications [2,3,4,5]. It is a highly branched polysaccharide made of short oligosaccharide side chains whose spatial arrangement in the amylopectin interior remains a topic of debate [6,7]. According to the well-established cluster model (CLM), the oligosaccharide side chains form double helices in the solid state that are arranged into clusters forming crystalline lamellae [8,9]. The clusters of short helices are connected to each other by longer oligosaccharide chains that run parallel to the orientation of the helices in the clusters. In 2004, Bertoft challenged the CLM and proposed the building block backbone model (BBBM), whereby 2–5 side chains are bundled in small groups coined building blocks [10]. The building blocks are connected to each other by longer oligosaccharides that run perpendicular to the orientation of the helical side chains. The evidence used in establishing the CLM or BBBM was mostly based on the enzymatic digestion of amylopectin combined with chromatographic analysis of the digested material [10,11,12,13,14,15,16]. However, a better understanding of the physical properties of amylopectin would benefit from further characterization of its constituting elements by non-destructive techniques, since bundles of oligosaccharide helices—whether building blocks or larger clusters—represent the fundamental constituting elements on which the CLM or BBBM are built. Such a study would require the application of non-invasive techniques to probe the interior of amylopectin.

Unfortunately, the characterization of the amylopectin interior is complicated by several physical constraints that hamper the use of most characterization techniques typically applied in macromolecular science. Its molecular weight is in the range of 10^7^ to 10^9^ g/mol, which makes it one of the largest naturally occurring macromolecules [11,17]. These large molecular weights lead to the large molecular dimension of amylopectin with average hydrodynamic diameters (*D*_h_) greater than 100 nm [18,19]. This feature prevents the use of gel permeation chromatography (GPC), since appropriate standards are not available to calibrate the GPC instrument for such a large macromolecule [18] which would merely be excluded from commercially available GPC columns whose *D*_h_ cut-off is around 50 nm [19]. Furthermore, the high polydispersity of amylopectin at all length scales complicates the use of scattering techniques which typically require monodisperse samples [20]. Finally, the classic fluorescence labeling techniques that target specific positions in a macromolecule [21,22] are not applicable for a macromolecule like amylopectin, since it is made of identical structural units that preclude its specific labeling, and its large size prevents the necessary photophysical crosstalk that occurs over a few nanometers between dyes and quenchers and which is required to retrieve information about their separation distance or the internal dynamics experienced by amylopectin.

In this context, the combination of pyrene excimer formation (PEF) [23]—between an excited and a ground-state pyrenyl label both covalently and randomly attached onto a macromolecule [24]—and the fluorescence *blob* model (FBM) to analyze the fluorescence decays of a macromolecule randomly labeled with pyrene, is ideally suited to study complex macromolecules like amylopectin [25,26]. The FBM acknowledges that a pyrenyl label cannot probe the entire macromolecular volume while it remains excited, but instead that its movement is restricted to a sub-macromolecular volume referred to as a *blob*. The *blob* can then be used as a unit volume to compartmentalize the entire macromolecular volume into a cluster of *blobs* among which the randomly attached pyrenyl labels distribute themselves according to a Poisson distribution. The FBM yields the number *N*_blob_^exp^ of structural units per *blob*, which represents the maximum number of structural units separating two pyrenyl labels while still allowing pyrene–pyrene encounters leading to PEF. In turn, molecular mechanics optimizations (MMOs) can be conducted on different conformations of a pyrene-labeled macromolecule to determine the theoretical *N*_blob_^theo^, which would match *N*_blob_^exp^ for the correct macromolecular conformation [27,28,29].

The first application of the PEF/FBM/MMO methodology to probe the interior of amylopectin and three nanosized amylopectin fragments—referred to as NAF(8), NAF(20), and NAF(56); with *D*_h_ equal to 8, 20, and 56 nm, respectively—led to the solution-cluster model (Sol-CLM), which suggests that in solution, the oligosaccharide helical side chains remain clustered inside amylopectin with an average interhelical distance (*d*_h-h_) of about 3.0 nm with the clusters being held together by longer oligosaccharide segments [29,30]. Since the Sol-CLM is a direct consequence of the CLM, these studies supported the CLM over the BBBM.

While insightful, these early PEF studies [29,30] did not characterize the number (NC) of oligosaccharide side chains per cluster. Herein, the PEF/FBM/MMO methodology is applied to measure NC for the four pyrene-labeled amylopectin-based polysaccharides (Py-A*b*PS)—namely Py-NAF(8), Py-NAF(20), Py-NAF(56), and Py-amylopectin. The PEF experiments identified building blocks made of 3–4 oligosaccharide helices which are assembled into increasingly larger clusters of helices upon increasing the osmotic pressure of the Py-A*b*PS dispersions by adding large amounts of unlabeled NAF(56). These findings suggest that, macroscopically, amylopectin in solution behaves like a highly branched macromolecule as expected from the Sol-CLM [29,30]; but also that, locally, the spatial arrangement of the oligosaccharide helices is well described by the BBBM [10,11]. Consequently, the arrangement of the oligosaccharide side chains of amylopectin presented in this study reconciles the predictions of the Sol-CLM, CLM, and BBBM.

## 2. Materials and Methods

*Materials*: EcoSynthetix (Burlington, ON, Canada) supplied three research-grade NAFs, that were prepared from waxy corn starch. The polysaccharides (PSs), which included the four A*b*PS samples (NAF(8), NAF(20), NAF(56), and amylopectin) and amylose, were handled in the same manner as described earlier [29,30,31]. The NAFs were purified by dialysis against water before being lyophilized. Amylopectin from waxy corn was purchased from Sigma Aldrich (Markham, ON, Canada) and precipitated in cold ethanol before use. All other chemicals were purchased from Sigma Aldrich (Markham, ON, Canada) and used without further purification.

*Preparation of pyrene-labeled PSs*: The procedure described earlier for the esterification of the polysaccharide hydroxyls with 1-pyrenebutyric acid (PyBA) to yield the pyrene-labeled PSs (Py-PSs), their purification, and their characterization was followed to the letter [29,30,31].

*Pyrene content determination*: A stock dispersion with a known concentration of Py-PS in DMSO was prepared before being diluted with different amounts of DMSO to change the Py-PS concentration. The absorbance of all Py-PS samples was kept lower than 2.0. The absorption spectra of the Py-PSs were acquired with a quartz cuvette having a 1.00 cm path length (*L*) with a Cary 100 bio-UV–vis spectrophotometer (Varian, Palo Alto, CA, USA). A straight line was obtained by plotting the absorbance as a function of the mass concentration of the dispersions. The slope (*m*) of the line equaled *ε_Py_* × *L* × *λ_Py_*, where *ε_Py_* is the molar absorption coefficient of the 1-pyrenebutyryl label equal to 41,400 M^−1^cm^−1^ at 346 nm in DMSO based on the *ε_Py_* of PyBA [29], *L* equaled 1.00 cm, and *λ_Py_* was the pyrene content in moles of pyrene per gram of Py-PS. The molar fraction (*x*) of pyrene-labeled AGUs in a given Py(*x*)-PS sample was determined by applying Equation (1), where *M_AGU_* and *M_Py_* are the molar mass of unlabeled and pyrene-labeled AGUs equal to 162 g/mol and 432 g/mol, respectively.

(1) $$x\;=\;\frac{M_{AGU}}{M_{AGU}\;-\;M_{Py}\;+\;\lambda_{Py}^{-1}}$$

The Py(100 × *x*)-PS samples used in this study were Py(5.5)-Amylose, Py(5.8)-NAF(8), Py(5.8)-NAF(20), Py(0.004)-NAF(56), Py(6.7)-NAF(56), and Py(4.2)-amylopectin.

*Preparation of Py(0.004)-NAF(56) dispersions:* Special care was applied to determine the lifetime (*τ*_M_) of the pyrenyl monomer, which is fixed in the fluorescence decay analysis of the Py-PS dispersions. To this end, the Py(0.004)-NAF(56) sample was prepared with an extremely low pyrene content to minimize intramolecular PEF. Very little PEF was detected up to a Py(0.004)-NAF(56) concentration of 13 wt % in DMSO, corresponding to a 3.2 mM pyrene concentration. More significant PEF was observed at Py(0.004)-NAF(56) concentrations above 13 wt % indicating intermolecular PEF. For this reason, unlabeled NAF(56) was mixed with Py(0.004)-NAF(56) to increase the starch concentration without inducing intermolecular PEF for dispersions with a NAF concentration higher than 13 wt %. In this concentration range, a stock solution was prepared by dispersing 4 wt % of Py(0.004)-NAF(56) in DMSO. Unlabeled NAF(56) (0.45–0.94 g) was added to 1.1–1.65 mL of the Py(0.004)-NAF(56) stock solution. The mixture was further diluted with DMSO to a final volume of 3.3 mL. At this stage, the mixture had a final concentration of less than 33 wt % of Py(0.004)-NAF(56) and NAF(56) combined. It contained enough solvent to disperse the solids. After a homogenous dispersion was obtained by stirring (Heidolph magnetic stirrer, MR Hei-Tec, Schwabach, Germany; the speed was within 100–1400 rpm and was adjusted to ensure vigorous mixing without spilling the solution), the DMSO was evaporated by flowing N_2_ over the dispersions and the polysaccharide content was determined. The dispersions were fluid enough to be transferred to a fluorescence cell to acquire their fluorescence spectra and decays. The dispersions were analyzed by steady-state (SSF) and time-resolved (TRF) fluorescence without degassing. The results from this study are reported as a function of the total concentration of Py(0.004)-NAF(56) and NAF(56) combined.

*Preparation of mixtures of pyrene-labeled polysaccharides and unlabeled NAF(56):* Dispersions of naked NAF(56) with a concentration smaller than 30 wt % were prepared by adding the required amount of unlabeled NAF(56) (0–30 wt %) to a Py-PS dispersion in DMSO with a pyrene concentration equal to 24 μM. The dispersions were stirred for two days at 60 °C, until they became homogeneous. For dispersions with a NAF(56) content greater than 30 wt %, the proper mass of NAF(56) was added to a dilute Py-PS dispersion in DMSO. DMSO was then evaporated so that the final dispersion would contain about 24 μM pyrene of the Py-NAF sample and a large excess of NAF(56), that would result in the desired starch concentration (>30 wt %).

*Intrinsic viscosity of amylopectin in DMSO*: The intrinsic viscosity of amylopectin in DMSO at 25 °C was determined with an Ubbelohde viscometer (Technical Glass Products, Gonzalez, CA, USA). Amylopectin was dispersed in DMSO by stirring the dispersion at 95 °C overnight. The dispersions were prepared with an amylopectin concentration ranging from 0.61 to 2.82 mg/mL.

*Steady-state fluorescence measurements*: The fluorescence emission spectra were acquired with a Photon Technology International LS-100 fluorometer equipped with a xenon arc lamp (PTI, London, ON, Canada). The fluorescence spectra of pyrene were obtained by exciting the dispersion at 346 nm and collecting the fluorescence intensity from 360 to 670 nm. Excitation and emission slit widths of 1.0 nm were used for the fluorescence experiments. The contributions of the pyrene monomer (*I*_M_) and excimer (*I*_E_) to the fluorescence spectra were quantified by integrating the fluorescence spectra from 376 to 382 nm for the monomer and from 500 to 530 nm for the excimer, respectively. The *I*_M_ and *I*_E_ fluorescence intensities were used to calculate the *I*_E_/*I*_M_ ratio, which provided a qualitative estimate of the PEF efficiency. The front-face geometry was applied when the concentration of a Py-A*b*PS dispersion was higher than 24 mM (equivalent to an absorption of 1.0 at 344 nm) to avoid the inner filter effect [22] at 379 nm corresponding to the 0–0 transition of pyrene [26]. Measurements conducted with the front face geometry were repeated three times.

**Figure 1:**
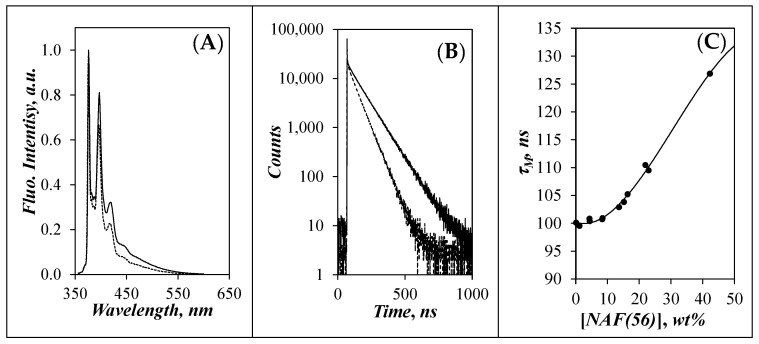
(**A**) Fluorescence spectra and (**B**) fluorescence decays of Py(0.004)-NAF(56) dispersions containing NAF(56) at a total [NAF(56)] of (---) 0.001 wt % and (―) 42.3 wt % in DMSO. (**C**) Plot of the lifetime (*τ*_M_) of Py(0.004)-NAF(56) as a function of total [NAF(56)] in DMSO.

*Time-resolved fluorescence measurements*: The time-resolved fluorescence decays were acquired with an IBH time-resolved fluorometer (IBH, Edinburgh, UK). The excitation wavelength was set at 346 nm with an excitation monochromator using a 340 nm nanoLED. A Ludox suspension was employed to obtain the instrument response function (IRF) by using a same excitation and emission wavelength of 346 nm. The fluorescence decays of the pyrene monomer and excimer were collected with a 1.02 ns/ch time-per-channel at 375 nm with a 370 nm cut-off filter and at 510 nm with a 495 nm cut-off filter, respectively. The cut off filters minimized the detection of light scattered by the PS dispersions. The fluorescence decays of two types of Py-PS dispersions were acquired. The short-lived emission of NAF(56) led to the appearance of a sharp spike at the beginning of the decays of the pyrenyl monomer in the Py(0.004)-NAF(56) dispersions (see Figure 1B). The decays were acquired with ~80,000 counts at the decay maximum to ensure that the decays would have at least 20,000 counts just after the spike to obtain a good signal-to-noise ratio in the longer-lived section of the decay. The fluorescence decays were fitted with a biexponential function, starting the analysis just after the spike. The exponential with the longest decay time had a pre-exponential contribution larger than 83% and its decay time was assigned to the natural lifetime of the pyrenyl monomer (*τ*_M_). All other dispersions of Py-PS had a much stronger pyrene fluorescence and the monomer and excimer decays of the pyrenyl labels were acquired with 20,000 counts at the decay maximum. These decays were fitted globally according to the fluorescence *blob* model (FBM).

*Global fluorescence blob model analysis*: The monomer and excimer fluorescence decays were fitted globally according to Equations (S1) and (S2) provided as Supplementary Material (SM) [29]. Within the FBM framework, five pyrene species can be found in a PyLM. *Py*_diff_* represents those pyrenyl labels, whose slow diffusion in solution is controlled by the motion of the AGUs, that *Py*_diff_* is bound to, and is described by the rate constant *k*_blob_. Once *Py*_diff_* comes near a ground-state pyrene, it turns into the species *Py*_k2_*, which undergoes a rapid rearrangement with a rate constant *k*_2_ to form an excimer *E*0* or EL* depending on whether the two pyrenyl species are well- or poorly stacked, respectively. Finally, those pyrenes located in the pyrene-poor regions of the macromolecule cannot form excimer and emit as if they were free in solution with the monomer lifetime *τ*_M_. They are referred to as *Py*_free_*. The molar fractions *f*_diff_, *f*_k2_, *f*_free_, *f*_E0_, and *f*_EL_ representing the species *Py*_diff_*, *Py*_k2_*, *Py*_free_*, E0*, and EL* can be retrieved from the FBM analysis of the fluorescence decays. The FBM analysis of the fluorescence decays also yields the average number <*n*> of pyrenyl labels per *blob*, which can be related to the number *N*_blob_^exp^ of structural units inside a *blob* according to Equation (2).

(2) $$N_{\mathrm{blob}}^{\exp}\;=\;\frac{1}{1-f_{\mathrm{Mfree}}}\times \frac{<n>}{x}$$

In Equation (2), *x* represents the molar fraction of AGUs bearing a pyrenyl label as defined by Equation (1) and *f*_Mfree_ represents the molar fraction of pyrenyl labels *Py*_free_* probed in the monomer decays. The parameters were optimized according to the Marquardt–Levenberg algorithm [32]. The fluorescence decay fits were deemed satisfactory, when the *χ*^2^ was smaller than 1.3 and the residuals and autocorrelation of the residuals were randomly distributed around zero as shown in Figure S1 in Supplementary Material. The parameters retrieved from all the decay analyses were listed in Tables S1–S10 in Supplementary Material.

## 3. Results

### 3.1. Size of Amylopectin and of the Nanosized Amylopectin Fragments

The number average hydrodynamic diameters (*D*_h_) of the three NAFs in DMSO were determined earlier by dynamic light scattering to equal 8, 20, and 56 nm and these NAFs were referred to as NAF(8), NAF(20), and NAF(56), respectively [29]. NAFs, also called starch nanoparticles (SNPs), are the focus of intense research [5,33,34,35,36]. The *D*_h_ of amylopectin was estimated to equal 227 (±20) nm from its intrinsic viscosity ([*η*]) equal to 122.5 (±0.3) mL/g as shown in Figure S2 in Supplementary Material and the relationship *D*_h_ = 0.0258 × [*η*]^1.89^, which was established earlier for a series of NAFs [29]. The values of *D*_h_, [*η*], and *M*_v_ obtained for all PSs are listed in Table 1.

It must be pointed out that the *M*_v_ values reported in Table 1 were obtained from the number average *D*_h_, which did not account for a small number of larger species in the polysaccharide dispersions [29]. Consequently, the *M*_v_ values in Table 1 are most likely underestimated. Nevertheless, they can be viewed as apparent molecular weights that show the expected trend of decreasing *M*_v_ with decreasing *D*_h_ for this series of polysaccharides.

### 3.2. Lifetime of the Pyrenyl Label as a Function of [NAF(56)]

The natural lifetime of the pyrene monomer (*τ*_M_) was estimated from the analysis of the TRF decays of Py(0.004)-NAF(56) in DMSO. The very low pyrene content of this sample ensured that no PEF occurred as illustrated by the absence of a broad structureless emission centered at 480 nm in Figure 1A, that would otherwise be characteristic of PEF. The fluorescence spectra and decays of Py(0.004)-NAF(56) were acquired in DMSO with increasing concentration of non-fluorescently labeled NAF(56). While the fluorescence spectra showed similar spectral features for Py(0.004)-NAF(56) in DMSO without and with enough NAF(56) to generate a total PS concentration of 42 wt %, the fluorescence decays shown in Figure 1B displayed some important differences. The spike observed at the beginning of the decay was attributed to the short intrinsic fluorescence of NAF(56) [37,38], while the longer contribution was due to the emission of isolated pyrenyl labels. The decays past the spike were fitted with a biexponential, where the exponential having the longest lifetime contributed more than 83% (Table S1) to the fluorescence decay and was attributed to *τ*_M_. *τ*_M_ was plotted as a function of the total concentration of Py(0.004)-NAF(56) and NAF(56) in Figure 1C.

*τ*_M_ was found to increase from 100.2 (±0.5) ns for NAF(56) concentrations smaller than 13 wt % before increasing progressively to 126.9 ns for a NAF(56) concentration of 42 wt %. *τ*_M_ for a pyrenyl label depends strongly on its accessibility to oxygen dissolved in the solvent, which was non-degassed DMSO in the present case. Based on the trend shown in Figure 1C, the increase in *τ*_M_ observed for a NAF(56) concentration larger than 13 wt % suggests that the diffusion of oxygen and the mobility of the pyrenyl labels are hindered at these large NAF(56) concentrations, resulting in the increase in *τ*_M_. In fact, the lifetime of 126.9 ns obtained for 42 wt % NAF(56) approaches that of 135 ns obtained for a pyrenyl label in non-aerated DMSO [29], suggesting that at this [NAF(56)], the viscosity of the dispersion is so high that it prevents oxygen from reaching an excited pyrenyl label.

### 3.3. Intraparticle Interaction

It was shown in earlier studies that the compression of Py-A*b*PSs can be induced by adding large quantities of poly(ethylene glycol) (PEG) [29,30]. Since the compression of Py-A*b*PSs induces an increase in the PEF efficiency, the magnitude of this increase was investigated to assess if it was compatible with a decrease in the interhelical distance *d*_h-h_ between the oligosaccharide helices in a cluster, or whether it involved the association of small building blocks into larger clusters. However, since the largest PEG sample used had a molecular weight of 20 K with an associated end-to-end distance (*r*_EE_) of ~12 nm [29,30], it might have been small enough to partially penetrate the A*b*PS interior. Addition of the much larger NAF(56) to a Py-A*b*PS dispersion was expected to minimize the possibility of NAF(56) penetrating the interior of a Py-A*b*PS sample. By monitoring the compression of a dilute Py-A*b*PS dispersion by PEF as a function of the concentration of unlabeled NAF(56), any change in fluorescence would be the result of intraparticle interaction due to the compression of the A*b*PSs induced by increased osmotic pressure. All PSs were labeled with about 5.9 (±0.6) mol % of pyrene, which resulted in a substantial amount of PEF even under dilute conditions, where PEF occurred intramolecularly. Since NAF(56) fluoresced, as reported earlier [37,38] and observed in Figure 1B, the concentration of the Py-PS samples was increased to prevent detection of the NAF(56) fluorescence, yet it remained sufficiently low to avoid interactions between the Py-PSs. As can be seen in Figure S3 in Supplementary Material, the *I*_E_/*I*_M_ ratio of Py(6.7)-NAF(56) remained constant for an absorbance of the pyrenyl labels in the Py(6.7)-NAF(56) dispersion between 0.1 and 1.5. The constancy of *I*_E_/*I*_M_ confirmed the absence of interactions between the Py(6.7)-NAF(56) samples over this range of absorbances. Consequently, fluorescence experiments were conducted with amylopectin, the three NAFs, and amylose labeled with 5.9 (±0.6) mol % pyrene using a pyrene absorbance for the Py-PS dispersions, that was close to unity in order to increase the fluorescence of the pyrenyl labels, while preventing Py-PS interactions. These conditions corresponded to a constant concentration of 2.4 × 10^−5^ mol/L of pyrenyl labels or 73 mg/L of Py-PS.

**Figure 2:**
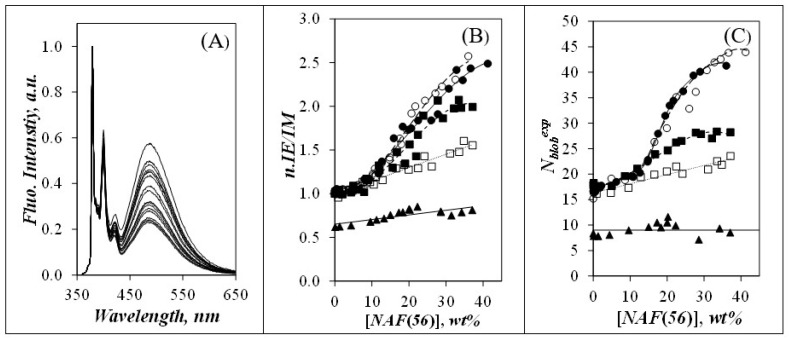
(**A**) Fluorescence spectra of Py(6.7)-NAF(56) acquired with increasing [NAF(56)] and normalized at 379 nm. (**B**) Plot of the normalized *I*_E_/*I*_M_ ratios. (**C**) Plot of *N*_blob_^exp^ as a function of [NAF(56)]. Symbols in (**B**,**C**): (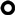) Py(4.2)-amylopectin, (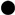) Py(6.7)-NAF(56), (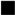) Py(5.8)-NAF(20), (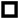) Py(5.8)-NAF(8), and (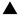) Py(5.5)-amylose.

As shown in Figure 2A for the Py(6.7)-NAF(56) sample in DMSO, the fluorescence of the excimer centered at 480 nm increased substantially relative to that of the pyrene monomer at 379 nm, as [NAF(56)] was increased from 0.0 to 36.0 wt %. Similar changes in the fluorescence spectra were observed for all Py-A*b*PS samples, but with different rates of PEF increase. These changes were better quantified by monitoring the *I*_E_*/I*_M_ ratio of the Py-PS dispersions as a function of [NAF(56)] in Figure 2B.

To account for the different pyrene contents for all the Py-PSs studied, the *I*_E_/*I*_M_-*vs*-pyrene content trends obtained in earlier publications [29,30,31] under dilute conditions were parametrized to predict the value of the *I*_E_/*I*_M_ ratios of the Py-PS samples for an arbitrary pyrene content of 5.5 mol %, corresponding to an *I*_E_/*I*_M_ ratio of 1.0 for Py-amylopectin. After this normalization, the increase in *I*_E_/*I*_M_ in Figure 2B was most pronounced for Py(4.2)-amylopectin and Py(6.7)-NAF(56), modest for Py(5.8)-NAF(20), fairly small for Py(5.8)-NAF(8), and hardly noticeable for Py(5.5)-Amylose. The *I*_E_/*I*_M_ ratio increased at a [NAF(56)] of 11 (±2) wt % for the Py-A*b*PSs excluding the Py(5.5)-Amylose sample, for which PEF remained almost unchanged. Changes in the *I*_E_/*I*_M_ ratio of pyrene-labeled macromolecules are best described by referring to Equation (3), which states that the *I*_E_/*I*_M_ ratio is proportional to the rate constant (*k*_diff_) for PEF by diffusive encounters between pyrenyl labels and the local concentration of ground-state pyrenes ([*Py*]_loc_) experienced by an excited pyrenyl label [29]. As discussed earlier, the increase in *τ*_M_ in Figure 1C reflected a decrease in the mobility of the pyrenyl groups at [NAF(56)] larger than 13 wt %, which hindered their encounters with oxygen molecules in the viscous dispersions. This decrease in mobility also implied that *k*_diff_ must have decreased for larger NAF(56) concentrations. Therefore, the increase in *I*_E_/*I*_M_ observed in Figure 2B for several Py-A*b*PSs could only be due to an increase in [*Py*]_loc_. As interpenetration of these highly branched PSs was unlikely, addition of NAF(56) must have led to an increase in osmotic pressure resulting in the compression of the Py-A*b*PSs which brought the helical side chains of the A*b*PSs closer to each other. In contrast, Py-Amylose being fully exposed to the solvent could not respond to the compression, [*Py*]_loc_ was unaffected, and *I*_E_/*I*_M_ remained more or less constant within experimental error.

(3) $$\frac{I_{E}}{I_{M}}\;\propto \;k_{\mathrm{diff}}\times {\left[Py\right]}_{\mathrm{loc}}$$

While analysis of the *I*_E_/*I*_M_ ratios demonstrated the compression of the Py-A*b*PS samples upon increasing [NAF(56)], the retrieved information remained qualitative. Quantitative information about the Py-PSs could be obtained through the FBM analysis of their fluorescence decays. The FBM analysis yielded *N*_blob_^exp^, which represents the maximum number of structural units separating two AGUs bearing a pyrenyl label, while still allowing PEF to occur. *N*_blob_^exp^ was obtained by introducing the parameters <*n*> and *f*_Mfree_ retrieved from the global FBM analysis of the monomer and excimer fluorescence decays into Equation (2). *N*_blob_^exp^ increased with increasing [NAF(56)] for all Py-PS samples except for Py(5.5)-Amylose in Figure 2C. After starting from a similar *N*_blob_^exp^ value of 16.7 (±1.1) averaged over all Py-A*b*PSs at low [NAF(56)], *N*_blob_^exp^ increased with increasing [NAF(56)], until it reached a maximum value in Figure 2C equal to 43, 40, 28, and 24 for Py(4.2)-amylopectin, Py(6.5)-NAF(56), Py(5.8)-NAF(20), and Py(5.8)-NAF(8), respectively. Since the increase in *N*_blob_^exp^ occurred at about the same [NAF(56)] in Figure 2C as that obtained for the increase in *I*_E_/*I*_M_ in Figure 2B, the increase in *N*_blob_^exp^ was attributed to PEF occurring between different helical oligosaccharide side chains whose interhelical distance (*d*_h-h_) was reduced due to an increase in osmotic pressure. As a result, the changes in *N*_blob_^exp^ reflect changes in *d*_h-h_ and the arrangement of the side chains of amylopectin in its interior, and consequently, it should be possible to analyze the changes in *N*_blob_^exp^ with [NAF(56)] to determine how *d*_h-h_ varies with [NAF(56)] for the Py-A*b*PS samples.

In an earlier study, molecular mechanics optimizations (MMOs) were conducted with HyperChem (Gainsville, FL, USA) on a hexagonal array of seven helices that were labeled with pyrene and whose *d*_h-h_ was varied to predict how many AGUs in the array could be labeled with pyrene and allow the pyrenyl labels to come into contact to form an excimer. This procedure yielded a theoretical *N*_blob_ (*N*_blob_^theo^) that varied with *d*_h-h_ [29]. In turn, matching *N*_blob_^theo^ with *N*_blob_^exp^ allowed to predict *d*_h-h_ in the clusters of helices of Py-amylopectin. In this earlier study, *N*_blob_^theo^ was calculated by considering the interactions between the pyrenyl labels attached onto the central helix of the seven-helix hexagonal array and the pyrenyl labels attached onto the six surrounding helices [29]. Since *N*_blob_^theo^ reflected how a pyrenyl label attached on the central helix sensed the pyrenyl labels attached onto six peripheral helices, the *N*_blob_^theo^-*vs*-*d*_h-h_ relationship represented the interactions experienced by the pyrenyl labels attached onto helices constituting an infinite array of hexagonally packed helices. *N*_blob_^theo^ was found to increase with decreasing *d*_h-h_, a reasonable trend since a smaller *d*_h-h_ results in an array where the helices—and thus the pyrenyl labels—are closer to each other, which yields more efficient PEF as observed in Figure 2B,C.

While this behavior could qualitatively rationalize the increase observed for the *N*_blob_^exp^ values in Figure 2C with increasing [NAF(56)], it could not explain two features of the plots. First, the maximum *N*_blob_^theo^ obtained by assuming an infinite array of helices was 37, a value that was lower than the *N*_blob_^exp^ values of 41 (±2) obtained for the Py-amylopectin and Py-NAF(56) samples in Figure 2C at high [NAF(56)]. Second, the maximum value taken by *N*_blob_^exp^ in Figure 2C depended on the size of the A*b*PSs, something that could not be accounted for by the earlier *N*_blob_^theo^-*vs*-*d*_h-h_ relationship [29]. These discrepancies were attributed to the fact that the earlier *N*_blob_^theo^-*vs*-*d*_h-h_ relationship for an infinite array of oligosaccharide helices might have been justified for large A*b*PSs such as amylopectin and NAF(56), but not for smaller A*b*PSs such as NAF(8) and NAF(20), whose smaller dimension might not accommodate the large number of helices that would reflect an infinite array of helices. These considerations led to the study of the effect that the size of a finite array of helices might have on the *N*_blob_^theo^-vs.-*d*_h-h_ relationship.

### 3.4. Finite Array of Helices

**Figure 3:**
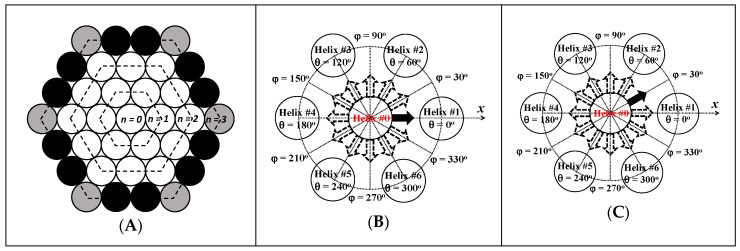
(**A**) Illustration of an array of helices of generation *n* = 3, where the helices are defined according to their location as (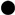) side, (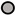) corner, and (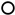) internal. The two configurations of pyrenyl labels considered in this study with the reference pyrenyl shown as a black arrow on the central Helix #0 located at (**B**) *φ* = 0° and (**C**) *φ* = 30° in a cluster of seven helices.

A hexagonal array of helices is depicted in Figure 3A, where the array expands outward from the central helix in a layer-by-layer fashion, with each layer being numbered from *n* = 0 for the central helix and increasing by one unit at a time for each additional concentric layer of helices. As an example, the array shown in Figure 3A would thus be a generation *n* = 3 array. The helices were classified into three different categories depending on their location and the associated number of adjacent helices, that they could interact with (see Table 2). A complete array of generation *n* is comprised of a total number *H* = 3*n^2^*+ 3*n* + 1 of helices, where 3*n*^2^ − 3*n* + 1 and 6*n* helices are internal and peripheral helices, respectively, as shown in Figure 3A. Since there are always six corner helices in the complete array, (6*n* − 6) helices are present on the six sides at the periphery of the hexagonal array.

The earlier *N*_blob_^theo^-*vs*-*d*_h-h_ relationship [29] was established by considering that the reference pyrenyl label in Helix #0 in Figure 3B was aiming toward Helix #1 along the *x*-axis defined by the line linking the centers of Helices #0 and #1. The orientation of the reference pyrene was defined by the angle *φ*, which equaled 0 in this earlier case [29]. The earlier *N*_blob_^theo^-*vs*-*d*_h-h_ relationship was refined in the present study by considering that the reference pyrene could also be oriented along the bisector of the angle defined by Helices #1, 0, and 2 in Figure 3C, corresponding to an angle *φ* equal to 30° with respect to the horizontal *x*-axis. This refinement allowed the study of the interhelical interactions for a reference pyrene on Helix #0 at 12 possible *φ* angles stemming from the central Helix #0 in 30° increments from 0° to 330°. Beside the angle *φ* defining the orientation of the reference pyrene on a given helix with respect to the *x*-axis, the position of a helix in the array was described by the angle *θ* between the line joining the centers of helix #0 and that of helices #1–6 and the *x*-axis as shown in Figure 3B,C.

**Figure 4:**
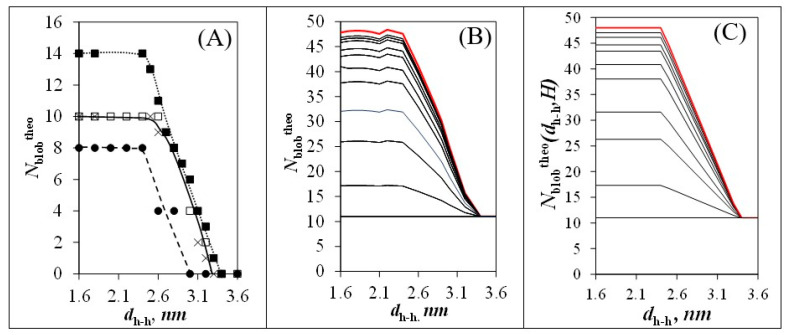
(**A**) Contributions to *N*_blob_ resulting from interhelical PEF when *j* equals 0° and *θ* equals (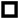) 0° and (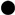) 60° and when *φ* equals 30° and *θ* equals (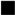) 60° and (×) 120° (see Figure 3B,C). (**B**) Plot of *N*_blob_^theo^ with arrays constituted of different total numbers (*H*_total_) of helices. From bottom to top: *H* = 1, 2, 4, 7, 19, 37, 169, 547, 1027, and **∞**. (**C**) Plot of *N*_blob_^theo^(*d*_h-h_,*H*) after parametrization according to Equations (5)–(7) for the same arrays of helices as in (**B**).

Since the MMOs had already been conducted earlier for a *φ* value of 0° [29], they were repeated herein for a reference pyrene having an orientation *φ* in Figure 3C equal to 30°. The secondary pyrenyl label was covalently attached to a second helix, separated from the central Helix #0 by a *d*_h-h_, which was adjusted from 1.6 to 3.4 nm in 0.1 nm increments. The primary and secondary pyrenyl groups were induced to overlap by conducting MMOs with HyperChem in the same manner as what had been carried out earlier [29]. In these MMOs, the pyrenyl moiety and its linker down to the oxygen of the C2-hydroxyl of the AGU to which the 1-pyrenebutyryl group was attached, was allowed to move, while the polysaccharide backbone of both helices was kept immobile. If the two frames of the overlapping pyrenyl labels retained a planar structure and resulted in seven or more carbons of the reference pyrenyl overlapping the frame of the secondary pyrenyl, the overlap was considered suitable for successful PEF. *N*_blob_^theo^ was then increased by one unit to account for the AGU bearing the secondary pyrenyl group leading to successful PEF. For a given *d*_h-h_, the MMOs were repeated by changing the position of the secondary pyrenyl group, along the backbone of the secondary helix, one AGU at a time, until the pyrenyl labels were too far apart to enable PEF. The MMOs were then repeated by changing *d*_h-h_ from 1.6 to 3.4 nm in 0.1 nm increments. *N*_blob_^theo^ of each helix with an orientation defined by an angle *θ* = 0° and 60° for *φ* equal to 0° as depicted in Figure 3B and at an angle *θ* = 60° and 120° for *φ* equal to 30° as depicted in Figure 3C was plotted as a function of *d*_h-h_ in Figure 4A. The MMO results are listed in Tables S11 and S12 as Supplementary Material. Over this range of *d*_h-h_ values, no interactions could be detected between a reference pyrene attached on Helix #0 and a secondary pyrenyl label attached on a helix located at an angle *θ* greater than or equal to 90° or 120° in Figure 3B or Figure 3C, where *φ* equaled 0° or 30°, respectively.

The effect of the position of the reference pyrene with different orientations in Helix #0 can be visualized in Figure 3B,C. In Figure 3B, the reference pyrene with *φ* = 0° can interact with the three adjacent helices at *θ* = 0°, 60°, and 300°, as reported earlier [29]. In Figure 3C, the reference pyrene with *φ* = 30° can interact with the four adjacent helices located at *θ* = 0°, 60°, 120°, and 300°. The *N*_blob_^theo^ (*φ* = 0°) or *N*_blob_^theo^ (*φ* = 30°) values obtained for a primary pyrene located on Helix #0 with an orientation *φ* of 0° or 30° would thus equal [*N*_blob_^intra^ + *N*_blob_ (*φ* = 0°, *θ* = 0°) + 2 × *N*_blob_ (*φ* = 0°, *θ* = 60°)] or [*N*_blob_^intra^ + 2 × *N*_blob_ (*φ* = 30°, *θ* = 60°) + 2 × *N*_blob_ (*φ* = 30°, *θ* = 120°)], respectively. In these expressions, *N*_blob_^intra^ represents the maximum number of AGUs, that would allow PEF between a reference and a secondary pyrenyl label both attached onto Helix #0. *N*_blob_^intra^ was determined earlier and found to equal 11 for a single helix with seven AGUs per turn [31]. In turn, *N*_blob_^theo^ (*φ* = 0°) and *N*_blob_^theo^ (*φ* = 30°) could be used to determine $N_{\mathrm{blob},\mathrm{internal}}^{\mathrm{theo}}$ for an internal helix, such as Helix #0 in Figure 3B,C, surrounded by six helices in the hexagonal array. The expression for $N_{\mathrm{blob},\mathrm{internal}}^{\mathrm{theo}}$ is provided in Table 2. A similar methodology was applied to determine the expressions for $N_{\mathrm{blob},\mathrm{side}}^{\mathrm{theo}}$ and $N_{\mathrm{blob},\mathrm{corner}}^{\mathrm{theo}}$ for helices located at a side or a corner in the hexagonal array shown in Figure 3A and their expressions were also listed in Table 2. Using the same procedure, *N*_blob_^theo^ for a complete *n*th generation array of helices could be determined with Equation (4).

(4) $$N_{\mathrm{blob}}^{\mathrm{theo}}\;=\;\frac{(3n^{2}-3n+1)N_{\mathrm{blob},\mathrm{internal}}^{\mathrm{theo}}+(6n-6)N_{\mathrm{blob},\mathrm{side}}^{\mathrm{theo}}+6N_{\mathrm{blob},\mathrm{corner}}^{\mathrm{theo}}}{3n^{2}+3n+1}$$

The trends given in Figure 4A for *N*_blob_^theo^ (*φ* = 0°, *θ* = 0°), *N*_blob_^theo^ (*φ* = 0°, *θ* = 60°), *N*_blob_^theo^ (*φ* = 30°, *θ* = 60°), and *N*_blob_^theo^ (*φ* = 30°, *θ* = 120°) as a function of *d*_h-h_ could then be applied to determine how *N*_blob_^theo^ would vary as a function of *d*_h-h_ and the number of helices included in a given array as shown in Figure 4B. The *N*_blob_^theo^ values merged into a single value equal to *N*_blob_^intra^ for *d*_h-h_ values larger than 3.6 nm, where PEF occurred between two pyrenyl labels attached to the same helix. Since considering the angle *φ* = 30° in the MMOs led to an increase in *N*_blob_^theo^, the cut off where PEF could only occur intra-helically was increased from 3.2 nm, when only *φ* = 0° was considered [29], to 3.6 nm with the present upgraded version of MMOs, which included *φ* = 0° and *φ* = 30°. As *d*_h-h_ decreased, *N*_blob_^theo^ increased and spread more widely with increasing number of helices in an array, reaching a maximum spread for a *d*_h-h_ value of 2.4 nm. Interestingly, the plot of *N*_blob_^theo^-*vs*-*d*_h-h_ in Figure 4B could be viewed as being the mirror image of the plot of *N*_blob_^exp^-*vs*-[NAF(56)] obtained for the Py-A*b*PS samples in Figure 2C. This striking resemblance suggested that *d*_h-h_ and [NAF(56)] must be related to each other.

To find a mathematical relationship between *d*_h-h_ and [NAF(56)], the following procedure was applied. First, the *N*_blob_^theo^-*vs*-*d*_h-h_ profiles were satisfyingly parametrized with the two functions *f*(*H*) and *g*(*d*_h-h_), which depended solely on the number *H* of helices in an array and the interhelical distance *d*_h-h_ and whose expressions are provided in Equations (4) and (6), respectively.

(5) $$f(H)\;=\;37\times \left(1-\frac{1.173}{\sqrt{H}}\right)$$

*g*(*d*_h-h_) = 1.0    for *d*_h-h_ ≤ 2.4 nm (6a)

*g*(*d*_h-h_) = (3.3715 − *d*_h-h_)/0.9587    for 2.4 nm ≤ *d*_h-h_ ≤ 3.3 nm (6b)

*g*(*d*_h-h_) = 0.0    for *d*_h-h_ ≥ 3.3 nm (6c)

Adding *N*_blob_^intra^ (= 11) to the product of the functions *f*(*H*) and *g*(*d*_h-h_) yielded a mathematical representation of *N*_blob_^theo^(*d*_h-h_,*H*) as shown in Equation (7), which provided an excellent representation for *N*_blob_^theo^ as can be seen through comparison of Figure 4B,C.

(7) $$N_{\mathrm{blob}}^{\mathrm{theo}}(d_{h-h},H)\;=\;f(H)\times g(d_{h-h})\;+\;11$$

**Figure 5:**
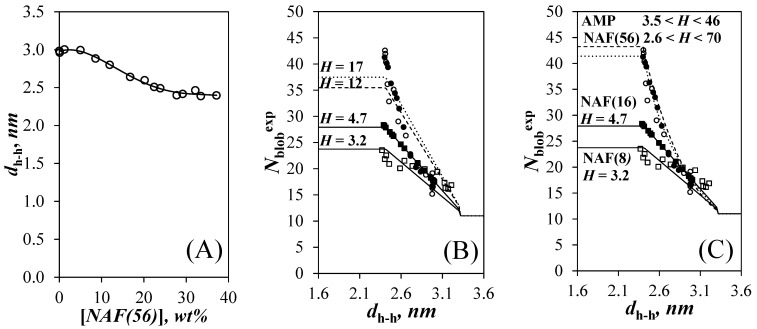
(**A**) Plot of *d*_h-h_ as a function of [NAF(56)] for the Py(5.8)-NAF(20) sample. Plots of *N*_blob_^exp^ as a function of *d*_h-h_ for (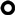) Py(4.2)-amylopectin, (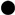) Py(6.7)-NAF(56), (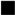) Py(5.8)-NAF(20), (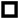) Py(5.8)-NAF(8), and (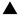) Py(5.5)-amylose for arrays with (**B**) a fixed number and (**C**) variable number of helices for the Py(6.7)-NAF(56) and Py(4.2)-amylopectin samples.

Out of all four *N*_blob_^exp^-*vs*-[NAF(56)] trends shown in Figure 2C, the trend with Py(5.8)-NAF(20) showed a well-defined plateau of 27.9 (±0.5) between [NAF(56)] of 28 and 33 wt %. The plateau region suggested that at high [NAF(56)], the oligosaccharide helices of Py(5.8)-NAF(20) were within *d*_h-h_ < 2.4 nm, where *N*_blob_^exp^ was not expected to change any longer according to the *N*_blob_^theo^-*vs*-*d*_h-h_ trends shown in Figure 4B. Under those conditions, *g*(*d*_h-h_) equals unity according to Equation (6a), which allowed the determination of *H* with Equation (7), found to equal 4.7. With *H* known for Py(5.8)-NAF(20), the relationship shown in Figure 5A could be obtained between *d*_h-h_ and [NAF(56)] by using Equation (7) to extract *d*_h-h_ from the *N*_blob_^exp^ values obtained for that sample in Figure 2C. The sigmoidal curve shown in Figure 5A could be fitted with the fourth order polynomial given in Equation (8), which allowed a direct relationship between *d*_h-h_ and [NAF(56)].

*d*_h-h_ = −1.609 × 10^−6^[NAF(56)]^4^ + 1.522 × 10^−4^[NAF(56)]^3^ − 4.348 × 10^−3^[NAF(56)]^2^ + 1.838 × 10^−2^[NAF(56)] + 2.976 (8)

Since *I*_E_/*I*_M_ and *N*_blob_^exp^ increased at the same [NAF(56)] for all Py-A*b*PSs in Figure 2B,C, respectively, the clusters of helices in the Py-A*b*PS samples were assumed to experience a same osmotic pressure at a given [NAF(56)] and thus share a same *d*_h-h_ for a given [NAF(56)], which could now be obtained with Equation (8). The equivalence between *d*_h-h_ and [NAF(56)] provided by Equation (8) led to Figure 5B, where the *N*_blob_^exp^ values for the Py-A*b*PS samples represented as a function of [NAF(56)] in Figure 2C could now be plotted as a function of *d*_h-h_. In order to obtain each *N*_blob_^exp^-*vs*-*d*_h-h_ profile shown in Figure 5B, Equation (8) was used to determine *d*_h-h_ for a given [NAF(56)]. With *d*_h-h_ known, the function *g*(*d*_h-h_) in Equation (6) could be determined, and the number *H* of oligosaccharide helices in a cluster could be extracted by equating *N*_blob_^exp^ to *N*_blob_^theo^ with Equation (7). After optimization of *H* for a set of [NAF(56)] and *N*_blob_^exp^ values obtained for a given Py-A*b*PS, that would result in a best match between *N*_blob_^theo^(*d*_h-h_,*H*) and *N*_blob_^exp^ at all *d*_h-h_, the clusters for Py(5.8)-NAF(8), Py(5.8)-NAF(20), Py(6.7)-NAF(56), and Py(4.2)-amylopectin were found to be made of a constant number *H* of oligosaccharide helices equal to 3.2, 4.7, 12, and 17, respectively. Based on these results, the larger A*b*PSs were constituted of larger clusters of helices, a reasonable result since the physical dimensions of the smaller NAF(8) and NAF(20) samples would not have been capable of hosting such large clusters of helices. However, the use of Equation (7) with a fixed *H* value for all [NAF(56)] obtained with a given Py = A*b*PS yielded rather poor fits for the *N*_blob_^exp^ values obtained for the Py(4.2)-amylopectin and Py(6.7)-NAF(56) samples in Figure 5B. One reason for this poor fit was that the *N*_blob_^exp^ values of all four Py-A*b*PS samples in Figure 2C were similar for [NAF(56)] between 0 and 15 wt % corresponding to *d*_h-h_ between 2.7 and 3.2 nm. This suggested that at these large *d*_h-h_, Py(4.2)-amylopectin and Py(6.7)-NAF(56) behaved as if they were constituted of clusters of helices that were as small as those of Py(5.8)-NAF(8) with *H* = 3.2 and Py(5.8)-NAF(20) with *H* = 4.7. However, as *d*_h-h_ decreased and approached 2.4 nm, *N*_blob_^exp^ increased up to 41 (±2) for Py(4.2)-amylopectin and Py(6.7)-NAF(56), an *N*_blob_^exp^ value that could only be reached if these larger polysaccharides were constituted of larger clusters of helices according to Figure 4B. These considerations led to the suggestion that as more NAF(56) was added to the Py-A*b*PS dispersions, small clusters of helices would come together to generate larger clusters of helices in the larger A*b*PS samples.

**Figure 6:**
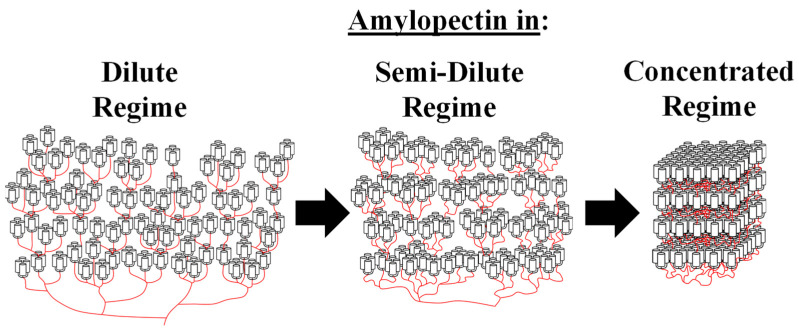
Re-arrangement of the building blocks into clusters of increasingly large size upon the compression of amylopectin.

**Figure 7:**
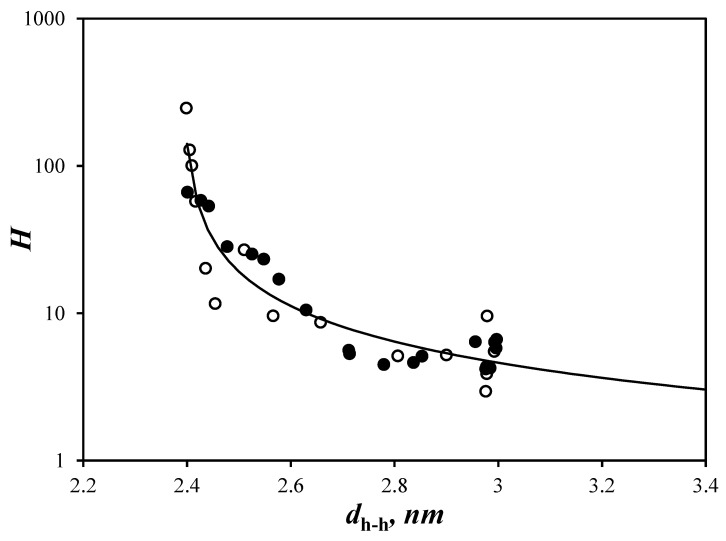
Plot of *H* as a function of *d*_h-h_ for (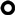) Py(4.2)-amylopectin and (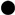) Py(6.7)-NAF(56).

Instead of assuming a constant number of helices per cluster, the *N*_blob_^exp^ values for Py(4.2)-amylopectin and Py(6.7)-NAF(56) were re-analyzed with Equation (7) to yield the *H* value at each *d*_h-h_. The agreement between *N*_blob_^exp^ and *N*_blob_^theo^(*d*_h-h_,*H*) was much improved in Figure 5C for Py(4.2)-amylopectin and Py(6.7)-NAF(56), when *H* was assumed to vary from 3.5 to 46 and from 2.6 to 70 for the former and latter A*b*PS, respectively. At large *d*_h-h_ values, the clusters were made of 3.2, 4.7, 2.6, and 3.5 oligosaccharide helices for Py(5.8)-NAF(8), Py(5.8)-NAF(20), Py(6.7)-NAF(56), and Py(4.2)-amylopectin, respectively, taking an average value of 3.5 (±0.9). These small clusters of helices were more likely the building blocks of the A*b*PS samples such as those evoked for the Building Block Backbone Model (BBBM) [10,11]. For the smaller Py(5.8)-NAF(8) and Py(5.8)-NAF(20) samples, the building blocks of helices must be isolated and cannot combine into larger clusters upon increasing the osmotic pressure of the dispersion by addition of NAF(56). The larger Py(6.7)-NAF(56) and Py(4.2)-amylopectin samples respond to the increase in osmotic pressure by combining several small building blocks into increasingly larger clusters to increase their internal density as depicted in Figure 6. The change in *H*-value upon increasing [NAF(56)] and decreasing *d*_h__−h_ is shown in Figure 7 for the Py(6.7)-NAF(56) and Py(4.2)-amylopectin samples. *H* was found to scale as 3.06 × (*d*_h-h_ − 2.39)^−0.832^, reflecting an exponential increase in cluster size with decreasing *d*_h-h_.

## 4. Discussion

The importance of clusters in the widely accepted CLM [7,8] led to an important scientific effort to characterize the size of the clusters found in amylopectin. Endoenzymes have been used to cleave long internal chains connecting clusters and thereby release the clusters, whose number of chains (NC) has been determined by light scattering [39], microscopy [40], chromatography [41], and exo-enzymatic treatment followed by ^1^H NMR analysis [42]. The NC constituting a cluster in amylopectin has been reported to range from as low as 4.2 side chains by light scattering [39] to as high as 17 double helices by microscopy [40], while chromatography measurements on enzymatically degraded amylopectin have yielded NC values between 8 and 19 [41,42,43,44]. In the more recent literature, NC values substantially lower than 8 have been attributed to the building blocks constituting the clusters of side chains [10,11,12,13]. Consequently, the NC values of 3.5 (±0.9) found by PEF for dilute Py-A*b*PS dispersions suggest that PEF probes the building blocks constituting the clusters of side chains. It is interesting that this NC value matched fairly well with the former estimate of 4.2 side chains per cluster obtained by light scattering measurements [39]. However, while the compression of NAF(8) and NAF(20) seems to result in isolated clusters that do not interact with each other—probably due to the small size of these A*b*PSs—compression of NAF(56) and amylopectin induced an increase in their cluster size. This conclusion was reached from the analysis of the *I*_E_/*I*_M_ and *N*_blob_^exp^ profiles obtained as a function of [NAF(56)] in Figure 2B,C, respectively. *I*_E_/*I*_M_ and *N*_blob_^exp^ were found to take similar values for all Py-A*b*PSs at [NAF(56)] smaller than about 13 wt %, but increased with increasing [NAF(56)]. Since both *I*_E_/*I*_M_ and *N*_blob_^exp^ respond to [*Py*]_loc_, their similar value obtained at low [NAF(56)] implied that all Py-A*b*PSs shared the same local concentration of side chains, and probably a similar building block size. This behavior changed at [NAF(56)] larger than 13 wt % with *I*_E_/*I*_M_ and *N*_blob_^exp^, showing marked differences between the Py-A*b*PSs. *I*_E_/*I*_M_ and *N*_blob_^exp^ were largest for Py(6.7)-NAF(56) and Py(4.2)-amylopectin, intermediate for Py(5.8)-NAF(20), and lowest for Py(5.8)-NAF(8). These differences could be rationalized by considering that, while the number of side chains per building block did not change for Py(5.8)-NAF(8) and Py(5.8)-NAF(20) with varying [NAF(56)], the small building blocks of ~3.5 side chains observed at low [NAF(56)] could assemble into larger clusters in Py(4.2)-amylopectin and Py(6.7)-NAF(56) made of several tens of side chains. It is possible that these larger arrays of oligosaccharide helices obtained at high [NAF(56)] are generated to increase the internal density of the A*b*PSs following an increase in osmotic pressure and that they are the precursors to the crystalline lamellae found in dry samples of amylopectin.

Amylopectin from waxy corn is constituted of oligosaccharides that are about 19 AGUs in length [41,45]. Consequently, a building block with 3.5 helices that are each 19 AGU-long would have an *M*_n_ of 1.07 × 10^4^ g/mol. The *M*_v_ of NAF(56), NAF(20), and NAF(8) were determined to equal 2.3 × 10^6^, 1.6 × 10^5^, and 1.8 × 10^4^ g/mol, respectively (see Table 1). NAF(8) and NAF(20) constituted of building blocks containing 3.2 and 4.7 helices would be expected to contain degraded clusters, where some helices were removed through debranching taking place during the degradation of amylopectin to produce the NAFs. Since the helices inside a building block are expected to be separated by ~3.3 nm in dilute dispersions, such a building block of 3.2 or 4.7 helices would have a size of between 3 and 6 nm, which would fit within the internal volume defined by the *D*_h_ of 8 and 20 nm for NAF(8) and NAF(20), respectively. However, the smaller dimension of NAF(8) and NAF(20) might make it more challenging to accommodate larger clusters of self-assembled building blocks upon compression induced by an increase in osmotic pressure. Consequently, the building blocks made of 3.5 oligosaccharide helices on average could be accommodated by all A*b*PSs studied in this report. PEF would mainly arise from the pyrene-labeled building blocks at low [NAF(56)] resulting in the same *I*_E_/*I*_M_ and *N*_blob_^exp^ values observed for all Py-A*b*PSs in Figure 2B,C, respectively. The increase in [NAF(56)] leads to an increase in osmotic pressure and the compression of the Py-A*b*PSs associated with the assembly of the building blocks into clusters of increasing size in the larger Py(6.7)-NAF(56) and Py(4.2)-amylopectin samples, resulting in a significant increase in *I*_E_/*I*_M_ and *N*_blob_^exp^ for these two samples.

The building block backbone model (BBBM) was proposed based on the experimental results of the enzymatic treatment of amylopectin [10,11]. According to the BBBM, clusters are constituted of several building blocks, which represent the ultimate branching unit, whose degree of branching varies from 2 to 10 for waxy corn amylopectin [10,11]. The building blocks are arranged perpendicularly to the backbone constituted of longer oligosaccharide chains. The side chains in the building blocks lead to the formation of the double helices, which constitute the crystalline lamellae of amylopectin in the solid state, while the long backbone chains run parallel to the lamellae and are located in the amorphous phase. The arrangement of the long backbone chains with respect to the side chains in the BBBM represents the main difference with the CLM. In polymer science, amylopectin described with the BBBM or CLM can be viewed as a bottle-brush [46,47] or hyperbranched polymer [48,49], respectively. The phenomenon of compression of amylopectin observed with the previous [29,30] and present studies suggest that amylopectin behaves like a hyperbranched polymer as predicted by the CLM. However, the continuous increase in cluster size observed in Figure 7 resulting in their probable arrangement into lamellae upon compression is more representative of the BBBM, whose natural arrangement of the building blocks along the long oligosaccharide chains forming the backbone would be more conducive of the self-assembly of the building blocks into increasingly larger clusters, as shown in Figure 6 [6]. Our results would thus suggest that the overall spatial arrangement of the oligosaccharide helices agrees with the CLM [8,9] and Sol-CLM [29], resulting in a hyperbranched polymeric architecture that can be compressed upon an increase in osmotic pressure, but that locally, the building blocks might be arranged perpendicularly along longer oligosaccharides that would result in the rapid increase in cluster size upon an increase in osmotic pressure as predicted by the BBBM [10,11]. Finally, if increasing the A*b*PS concentration leads to the self-association of the building blocks into clusters of increasing size as suggested by the experiments presented in this report, the study of A*b*PSs by different techniques requiring different ranges of A*b*PS concentrations depending on their respective detection limits would be probing clusters of different sizes, which might explain the range of cluster sizes reported in the literature [10,11,39,40,41,42].

## 5. Conclusions

The compressibility of samples of amylopectin and NAFs was studied by monitoring the *I*_E_/*I*_M_ ratio and *N*_blob_^exp^ as a function of [NAF(56)]. The increase in [NAF(56)] above 12 (±1) wt % increased the osmotic pressure in the dispersions, which triggered the compression of these branched A*b*PSs. The compression reduced the *d*_h-h_ resulting in an increase in [*Py*]_loc_, which translated into larger *I*_E_/*I*_M_ and *N*_blob_^exp^ values as [NAF(56)] increased. Amylopectin and the three NAFs showed evidence of compression at 12 (±1) wt % regardless of their size. The maximum values reached by the *I*_E_/*I*_M_ ratio and *N*_blob_^exp^ upon increasing [NAF(56)] showed a dependency with the size of the A*b*PSs. Py(4.2)-amylopectin and Py(6.7)-NAF(56) showed the largest increase in *N*_blob_^exp^, followed by Py(5.8)-NAF(20), and finally Py(5.8)-NAF(8). These observations suggested that PEF between pyrenyl labels was generated in finite domains, assumed to be clusters of helices, whose size increased upon compression following an increase in osmotic pressure induced by increasing [NAF(56)]. MMOs were conducted to predict how *N*_blob_^theo^ would vary as a function of the number of helices in a cluster and as a function of *d*_h-h_. Comparison of *N*_blob_^theo^ with *N*_blob_^exp^ led to the conclusion that the constituting elements of all A*b*PSs were similarly sized building blocks of 3.5 (±0.9) side chains, but that an increase in osmotic pressure induced by an increase in [NAF(56)] led to the self-assembly of the building blocks into clusters, whose size continuously increased for Py(4.2)-amylopectin and Py(6.7)-NAF(56) with increasing [NAF(56)]. The small constituting building blocks of ~3.5 side chains were common to all the A*b*PSs and self-assembled into larger arrays of helices, where the oligosaccharide helices would be packed more efficiently to reduce their overall size to accommodate the increase in osmotic pressure. Considering how important the notion of clusters of helices is in amylopectin and A*b*PSs derived from it, the ability to probe the size of the building blocks leading to the formation of these clusters on intact amylopectin molecules by PEF experiments is expected to find numerous applications for the characterization of amylopectin.

## Figures and Tables

**Table 1:** Hydrodynamic diameter (*D*_h_), intrinsic viscosity ([*η*]), viscosity (*M*_v_) average molecular weight of the polysaccharide samples, which were determined earlier [29].

Sample	*D*_h_ (nm) ^a^	[*η*] ^b^ (mL/g)	*M*_v_ (g/mol) ^c^
Amylopectin	227	122.5	7.2 × 10^7^
NAF(56)	56	61	2.3 × 10^6^
NAF(20)	20	40	1.6 × 10^5^
NAF(8)	8	23	1.8 × 10^4^

^a^. Number average hydrodynamic diameter in DMSO determined with DLS. ^b^. [*η*] was determined at 25 °C in DMSO. ^c^. The *M*_v_ were calculated with the calibration curve determined with number average *D*_h_ and [*η*].

**Table 2:** Illustration of a helix surrounded by different number of adjacent helices and its corresponding *N*_blob_.

Name (No. Adjacent Helices)	Arrangement	Expressions of *N*_blob_^theo^ for Different Arrays of Helices
Single (1)	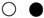	$\frac{12N_{\mathrm{blob}}^{\mathrm{intra}}+N_{\mathrm{blob}}(0,0)+2N_{\mathrm{blob}}(30,60)+2N_{\mathrm{blob}}(0,60)+2N_{\mathrm{blob}}(30,120)}{12}$
Vertex (2)	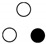	$\frac{12N_{\mathrm{blob}}^{\mathrm{intra}}+2N_{\mathrm{blob}}(0,0)+4N_{\mathrm{blob}}(30,60)+4N_{\mathrm{blob}}(0,60)+3N_{\mathrm{blob}}(30,120)}{12}$
Corner (3)	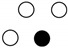	$\frac{12N_{\mathrm{blob}}^{\mathrm{intra}}+3N_{\mathrm{blob}}(0,0)+6N_{\mathrm{blob}}(30,60)+6N_{\mathrm{blob}}(0,60)+6N_{\mathrm{blob}}(30,120)}{12}$
Side (4)	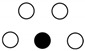	$\frac{12N_{\mathrm{blob}}^{\mathrm{intra}}+4N_{\mathrm{blob}}(0,0)+8N_{\mathrm{blob}}(30,60)+6N_{\mathrm{blob}}(0,60)+8N_{\mathrm{blob}}(30,120)}{12}$
Quasi-Internal (5)	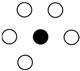	$\frac{12N_{\mathrm{blob}}^{\mathrm{intra}}+5N_{\mathrm{blob}}(0,0)+10N_{\mathrm{blob}}(30,60)+10N_{\mathrm{blob}}(0,60)+10N_{\mathrm{blob}}(30,120)}{12}$
Internal (6)	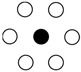	$N_{\mathrm{blob}}^{\mathrm{intra}}+\frac{N_{\mathrm{blob}}(0,0)}{2}+N_{\mathrm{blob}}(30,60)+N_{\mathrm{blob}}(0,60)+N_{\mathrm{blob}}(30,120)$

## Supplementary Materials

The following supporting information can be downloaded at: https://www.mdpi.com/article/10.3390/polym14163418/s1, Equations used in the FBM analysis of the fluorescence decays; example of decay fit; intrinsic viscosity measurements for amylopectin; plot of *I*_E_/*I*_M_ as a function of the absorbance of Py(6.7)-NAF(56) dispersions in DMSO; list of parameters retrieved from the fluorescence decay analysis; results from the MMOs. Figure S1. Example of the fit of the fluorescence decays of the pyrene monomer (left, *λ*_em_ = 375 nm) and excimer (right, *λ*_em_ = 510 nm) for Py(4.2)-Amylopectin labeled with 4.2 mol% of PyBA in DMSO. *χ*^2^ = 1.16, *λ*_ex_ = 346 nm; Figure S2. Plot of the (○) reduced and (●) inherent viscosity of amylopectin in DMSO at 25 °C as a function of amylopectin concentration; Figure S3. Plot of the *I*_E_/*I*_M_ ratio as a function of absorbance for Py(6.7)-NAF(56) in DMSO.; Table S1. Parameters retrieved from the fluorescence decays of Py(0.004)-NAF(56) at different concentrations of NAF(56) and Py(0.004)-NAF(56) analysed with the biexponential function given in Equation (S15); Table S2. Parameters retrieved from the global FBM analysis of the monomer decays with the program *globmis90sbg-2* for Py(4.1)-Amylopectin and Py(6.6)-NAF(56) at different NAF(56) concentrations in DMSO with Equations (S1) and (S2); Table S3. Parameters retrieved from the global FBM analysisof the monomer decays with the program *globmis90sbg-2* for Py(5.8)-NAF(20) and Py(5.8)-NAF(8) at different NAF(56) concentrations in DMSO with Equations (S1) and (S2); Table S4. Parameters retrieved from the global FBM analysis with the program *globmis90sbg-2* for the excimer decays of Py(4.1)-Amylopectin and Py(6.6)-NAF(56) at different NAF(56) concentrations in DMSO with Equations (S1) and (S2). τ_ES_ was fixed at 3.5 ns in the analysis; Table S5. Parameters retrieved from the global FBM analysis with the program *globmis90sbg-2* for the excimer decays of Py(5.7)-NAF(20) and Py(5.8)-NAF(8) at different NAF(56) concentrations in DMSO with Equations (S1) and (S2). τ_ES_ was fixed at 3.5 ns in the analysis; Table S6. Molar fractions of the pyrene species of Py(4.1)-Amylopectin(220) and Py(6.6)-NAF(56) at different NAF(56) concentrations in DMSO calculated from *f*_Mfree_, *f*_Mdiff_, *f*_Ediff_, *f*_k2_ and *f*_E0_; Table S7. Molar fractions of the pyrene species of Py(5.7)-NAF(20) and Py(5.8)-NAF(8) at different NAF(56) concentrations in DMSO calculated from *f*_Mfree_, *f*_Mdiff_, *f*_Ediff_, *f*_k2_ and *f*_E0_; Table S8. Parameters retrieved from the global FBM analysis with the program *globmis90sbg-2* for the monomer decays of Py(6.6)-NAF(56) at different Py(6.6)-NAF(56) concentrations in DMSO with Equations (S1) and (S2); Table S9. Parameters retrieved from the global FBM analysis with the program *globmis90sbg-2* for the excimer decays of Py(6.6)-NAF(56) at different Py(6.6)-NAF(56) concentrations in DMSO with Equations (S1) and (S2). τ_ES_ was fixed at 3.5 ns in the analysis; Table S10. Molar fractions of the pyrene species of Py(6.6)-NAF(20) at different Py(6.6)-NAF(56) concentrations in DMSO calculated from *f*_Mfree_, *f*_Mdiff_, *f*_Ediff_, *f*_k2_ and *f*_E0_; Table S11. Number of overlapping carbons between the reference pyrene attached on Helix #0 with an angle *φ* = 30° and the secondary pyrene attached on Helix 2 at θ = 60° for *d*_h-h_ values ranging from 3.4 nm to 1.6 nm with the total number of AGUs (Δ*N*_blob_) allowing a good pyrene overlap with 7 or more carbon atoms.; Table S12. Number of overlapping carbons between the reference pyrene attached on Helix #0 with an angle *φ* = 30° and the secondary pyrene attached on Helix 3 at θ = 120° for *d*_h-h_ values ranging from 3.4 nm to 1.6 nm with the total number of AGUs (Δ*N*_blob_) allowing a good pyrene overlap with 7 or more carbon atoms.
