# Supplementary material for: Cluster Size of Amylopectin and Nanosized Amylopectin Fragments Characterized by Pyrene Excimer Formation
Source: Polymers (Basel). 2022 Aug 21;14(16):3418. doi: 10.3390/polym14163418 (PMC9412863; doi:10.3390/polym14163418)
Supplement: Supplementary file 1 [file polymers-14-03418-s001.zip › polymers-1843290-supplementary.pdf]

Supplementary Material for

Cluster Size of Amylopectin and Nanosized Amylopectin Fragments Characterized by Pyrene Excimer Formation

Damin Kim and Jean Duhamel*

Department of Chemistry, Institute for Polymer Research, Waterloo Institute for Nanotechnology, University of Waterloo, Waterloo, ON N2L 3G1, Canada

* To Whom correspondence should be addressed.

Table of Content

A] Equations used in the global FBM analysis of the fluorescence decays ………………. 2-4

B] Example of decay fit……………………………………………………………….. 5

C] Intrinsic viscosity measurements for amylopectin ……………………………………… 5

D] I_E/I_M ratio as a function of the absorbance of Py(6.7)-NAF(56) dispersions in DMSO …. 6

E] List of parameters retrieved from the fluorescence decay analysis………………………. 6-14

F] Results from the MMOs ……………………………………………………………… 15-17

A] Global analysis of the monomer and excimer fluorescence decays according to the fluorescence blob model and equations for the molar fractions of the different pyrene species

The fluorescence decays of the pyrene monomer and excimer of the Py-NAFs and Py-Amylopectin samples were fitted with Equations S1 and S2, respectively. Equation S2 includes the existence of a short-lived excimer species, referred to as *ES**. The *ES** species is often encountered, when PEF takes place in rigid environments such as along the oligosaccharide backbone. Excellent fits were obtained with χ^2 values smaller than 1.3 and residuals and autocorrelation of the residuals randomly distributed around zero as shown in Figure S1.

$$[Py^*]_{(t)} = [Py^*_{diff}]_{(t)} + [Py^*_{k2}]_{(t)} + [Py^*_{free}]_{(t)}$$

$$= [Py^*_{diff}]_0 \exp\left(-\left(A_2 + \frac{1}{\tau_M}\right)t - A_3\left(1-\exp(-A_4 t)\right)\right)$$

$$+\left([Py^*_{k2}]_o + [Py^*_{diff}]_o e^{-A_3}\sum_{i=0}^{\infty}\frac{A_3^i}{i!}\frac{A_2+iA_4}{A_2+iA_4-k_2}\right)\exp\left(-\left(k_2+\frac{1}{\tau_M}\right)t\right)$$

$$-[Py^*_{diff}]_o e^{-A_3}\sum_{i=0}^{\infty}\frac{A_3^i}{i!}\frac{A_2+iA_4}{A_2+iA_4-k_2}\exp\left(-\left(A_2+iA_4+\frac{1}{\tau_M}\right)t\right) + [Py^*_{free}]_o \exp(-t/\tau_M)$$

(S1)

$$[E^*]_{(t)} = [E0^*]_{(t)} + [ES^*]_{(t)}$$

$$= k_2\left([Py^*_{k2}]_o + [Py^*_{diff}]_o e^{-A_3}\sum_{i=0}^{\infty}\frac{A_3^i}{i!}\frac{A_2+iA_4}{A_2+iA_4-k_2}\right)\times\frac{\exp\left(-\frac{t}{\tau_{E0}}\right)-\exp\left(-\left(k_2+\frac{1}{\tau_M}\right)t\right)}{k_2+\frac{1}{\tau_M}-\frac{1}{\tau_{E0}}}$$

$$+[Py^*_{diff}]_o e^{-A_3}\sum_{i=0}^{\infty}\frac{A_3^i}{i!}\frac{A_2+iA_4}{A_2+iA_4-k_2}\frac{\exp\left(-\left(A_2+iA_4+\frac{1}{\tau_M}\right)t\right)-\exp\left(-\frac{t}{\tau_{E0}}\right)}{A_2+iA_4+\frac{1}{\tau_M}-\frac{1}{\tau_{E0}}}$$

$$+[E0^*]_o \exp\left(-\frac{t}{\tau_{E0}}\right) + [ES^*]_o \exp\left(-\frac{t}{\tau_{ES}}\right) \quad \text{(S2)}$$

The parameters, A_2, A_3, and A_4 in Equation S1 and S2 are expressed in Equations S3a-c.

$$A_2 = \langle n \rangle \times \frac{k_{blob} k_e [blob]}{k_{blob} + k_e [blob]} \quad \text{(S3a)}$$

$$A_3 = \langle n \rangle \times \left(\frac{k_{blob}}{k_{blob} + k_e [blob]} \right)^2 \quad \text{(S3b)}$$

$$A_4 = k_{blob} + k_e \times [blob] \quad \text{(S3c)}$$

$\langle n \rangle$ in Equation S3b represents the average number of ground-state pyrenyl labels present in a *blob*. k_{blob} and k_{e} describe the rate constant for the slow diffusion of two AGUs bearing a pyrenyl label in a same *blob* and the rate constant for the exchange of ground-state pyrenes between *blobs*, respectively. [*blob*] is the local concentration of *blobs* inside the macromolecular volume.

The fluorescence *blob* model (FBM) was first applied to a series of pyrene-labeled amylopectin and NAFs with floating k_2, the rate constant for the rapid rearrangement between two pyrenyl labels in close proximity prior to PEF. The k_2 values of the pyrene-labeled amylopectin and NAF samples obtained at different NAF(56) concentrations were averaged. The average k_2 values obtained for each polysaccharide are listed in section E. The FBM analysis of the decays was repeated with the average k_2 value fixed in the analysis.

Equations S4-S6 provide the expressions for the molar fractions of the pyrene species determined from the analysis of the monomer decays. The molar fractions f_{Mdiff}, f_{Mfree}, and f_{Mk2} are expressed in Equation S4-S6, where all parameters have been defined in the main text.

$$f_{Mdiff} = \frac{[Py_{diff}^{*}]_o}{[Py_{diff}^{*}]_o + [Py_{k2}^{*}]_o + [Py_{free}^{*}]_o} \quad \text{(S4)}$$

$$f_{Mfree} = \frac{[Py_{free}^{*}]_o}{[Py_{diff}^{*}]_o + [Py_{k2}^{*}]_o + [Py_{free}^{*}]_o} \quad \text{(S5)}$$

$$f_{Mk2} = \frac{[Py_{k2}^{*}]_o}{[Py_{diff}^{*}]_o + [Py_{k2}^{*}]_o + [Py_{free}^{*}]_o} \quad \text{(S6)}$$

The molar fraction f_{Mfree} determined with Equation S5 was used to calculate the number of structural units present in a *blob* ($N_{\text{blob}}^{\text{exp}}$) as described by Equation 3 in the main text.

Similarly, the molar fractions of the pyrene species detected in the excimer decays was retrieved by applying Equation S2. The expressions of the molar fractions f_{Ediff}, f_{Ek2}, f_{EE0}, and f_{EES} are given in Equations S7-S10.

$$f_{Ediff} = \frac{[Py_{diff}^{*}]_o}{[Py_{diff}^{*}]_o + [Py_{k2}^{*}]_o + [E0^{*}]_o + [ES^{*}]_o} \quad \text{(S7)}$$

$$f_{Ek2} = \frac{[Py_{k2}^{*}]_o}{[Py_{diff}^{*}]_o + [Py_{k2}^{*}]_o + [E0^{*}]_o + [ES^{*}]_o} \quad \text{(S8)}$$

$$f_{EE0} = \frac{[E0^{*}]_o}{[Py_{diff}^{*}]_o + [Py_{k2}^{*}]_o + [E0^{*}]_o + [ES^{*}]_o} \quad \text{(S9)}$$

$$f_{EES} = \frac{[ES^{*}]_o}{[Py_{diff}^{*}]_o + [Py_{k2}^{*}]_o + [E0^{*}]_o + [ES^{*}]_o} \quad \text{(S10)}$$

The contribution of each pyrene species in the monomer and excimer decays were combined to determine the molar fraction of f_{diff}, f_{k2}, f_{free}, and f_{E0} as shown in Equation S11-S14. Since the short-lived species *ES** in Equation S2 emitted with a short decay time of 3.5 ns and disappeared rapidly after excitation, the contribution of the short-lived species was not included in the derivation of the molar fractions.

$$f_{diff} = \frac{1}{1 + (f_{Mk2}/f_{Mdiff}) + (f_{Mfree}/f_{Mdiff}) + (f_{EE0}/f_{Ediff})} \quad \text{(S11)}$$

$$f_{k2} = f_{diff}\,\frac{f_{Mk2}}{f_{Mdiff}} \quad \text{(S12)}$$

$$f_{free} = f_{diff}\,\frac{f_{Mfree}}{f_{Mdiff}} \quad \text{(S13)}$$

$$f_{E0} = f_{diff}\,\frac{f_{EE0}}{f_{Ediff}} \quad \text{d(S14)}$$

B] Example of a fit of the fluorescence decays

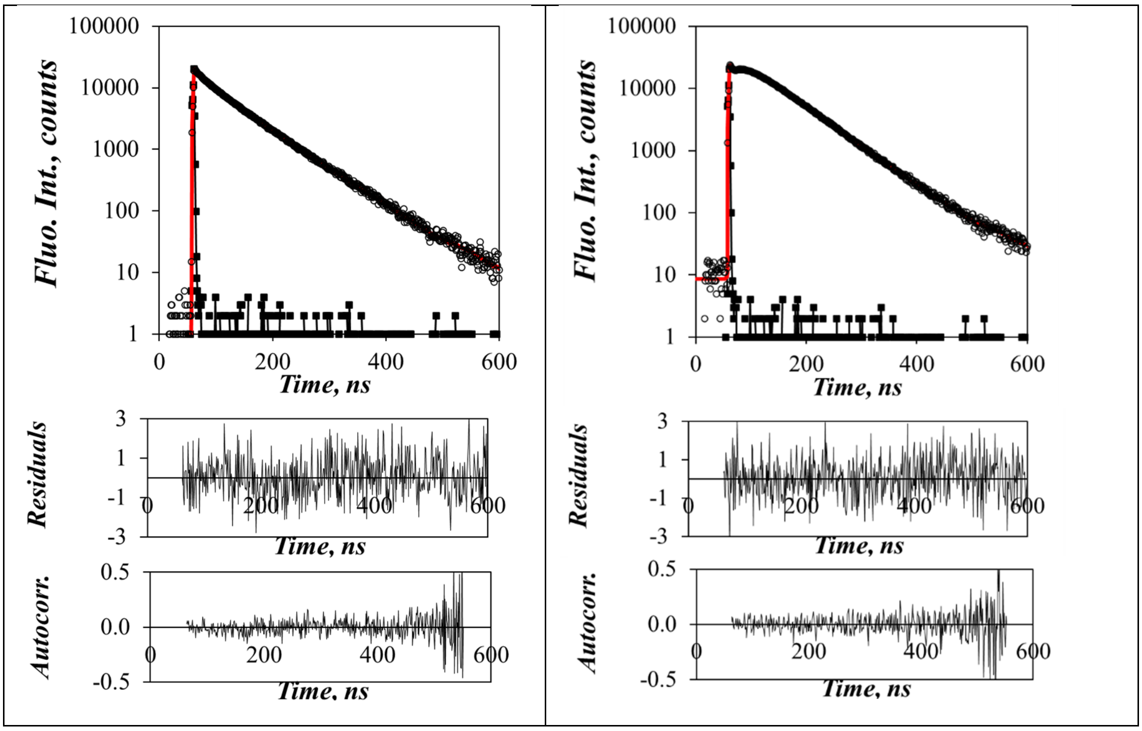

Figure S1. Example of the fit of the fluorescence decays of the pyrene monomer (left, λ_{em} = 375 nm) and excimer (right, λ_{em} = 510 nm) for Py(4.2)-Amylopectin labeled with 4.2 mol% of PyBA in DMSO. χ^2 = 1.16, λ_{ex} = 346 nm.

C] Plot of η as a function of mass concentration of amylopectin

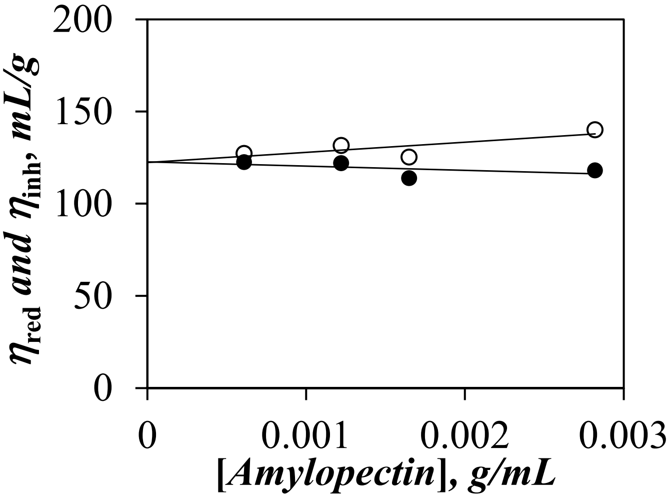

Figure S2. Plot of the (○) reduced and (●) inherent viscosity of amylopectin in DMSO at 25 °C as a function of amylopectin concentration.

D] Observation of a constant I_E/I_M ratio over a wide absorbance range for Py(6.7)-NAF(56)

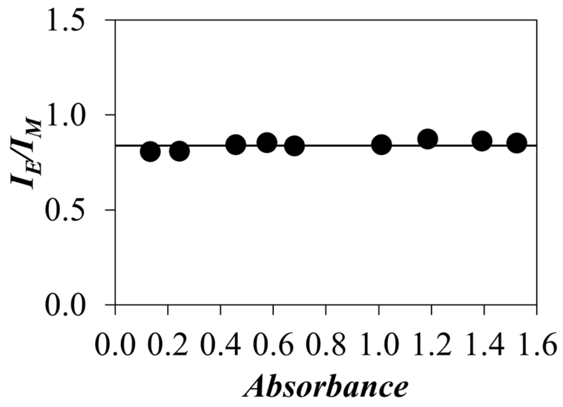

Figure S3. Plot of the I_E/I_M ratio as a function of absorbance for Py(6.7)-NAF(56) in DMSO.

E] List of parameters retrieved from the fluorescence decay analysis

Table S1. Parameters retrieved from the fluorescence decays of Py(0.004)-NAF(56) at different concentrations of NAF(56) and Py(0.004)-NAF(56) analysed with the biexponential function given in Equation S15.

$$[Py^*]_{(t)} = \alpha_1 \times \exp(-t/\tau_1) + \alpha_2 \times \exp(-t/\tau_2) \quad \text{(S15)}$$

wt %	α_1	τ_1 (ns)	α_2	τ_2 (ns)	$\chi 2$
0.1	0.14	20.3	0.86	100.1	1.13
1.2	0.15	25.9	0.85	99.5	1.11
4.3	0.13	31.6	0.87	100.8	1.08
4.4	0.15	25.9	0.85	100.4	1.09
8.3	0.15	23.5	0.85	100.7	1.02
8.4	0.14	27.8	0.86	100.9	1.17
13.6	0.15	23.6	0.85	102.9	1.08
15.2	0.15	24.6	0.85	103.8	1.11
16.3	0.14	22.3	0.86	105.2	1.09
21.9	0.15	23.3	0.85	110.5	1.14
23.0	0.15	29.3	0.85	109.5	1.20
42.3	0.17	26.5	0.83	126.9	1.06

Table S2. Parameters retrieved from the global FBM analysis of the monomer decays with the program *globmis90sbg-2* for Py(4.1)-Amylopectin and Py(6.6)-NAF(56) at different NAF(56) concentrations in DMSO with Equations S1 and S2.

Sample	*wt* %	f_{Mdiff}	f_{Mfree}	k_{blob} ($\times 10^7 s^{-1}$)	$<n>$	f_{k2}	$k_e[blob]$ ($\times 10^6 s^{-1}$)	$\chi 2$
Py(4.1)-Amylopectin (220) k_2=2.1×10^8 s^{-1}	0.0	0.841	0.047	1.488	0.65	0.112	9.27	1.16
	0.1	0.835	0.062	1.547	0.71	0.104	10.42	1.11
	1.2	0.836	0.044	1.405	0.74	0.120	9.87	1.17
	4.9	0.825	0.050	1.092	0.82	0.125	10.92	1.13
	8.6	0.802	0.040	1.311	0.81	0.158	10.36	1.05
	11.8	0.785	0.041	1.250	0.88	0.175	10.81	1.07
	16.6	0.771	0.036	1.036	1.12	0.193	9.32	1.12
	19.9	0.751	0.040	0.917	1.24	0.210	8.45	1.17
	22.5	0.731	0.035	0.862	1.50	0.234	7.81	1.10
	26.0	0.724	0.072	0.893	1.45	0.204	7.09	1.21
	27.8	0.685	0.060	0.820	1.58	0.255	6.92	1.11
	30.8	0.681	0.074	0.766	1.78	0.245	6.79	1.22
	32.8	0.647	0.059	0.751	1.83	0.294	5.46	1.23
	34.5	0.673	0.081	0.784	1.89	0.247	6.67	1.09
	36.6	0.646	0.071	0.774	1.93	0.282	7.58	1.09
	*41.3	0.607	0.107	0.835	2.00	0.287	9.53	1.12
Py(6.6)-NAF(56) k_2=2.0×10^8 s^{-1}	0.0	0.826	0.006	1.327	1.10	0.168	9.89	1.17
	0.1	0.821	0.003	1.345	1.11	0.176	10.03	1.21
	0.5	0.817	0.014	1.374	1.10	0.170	10.22	1.25
	1.3	0.805	0.007	1.249	1.17	0.189	9.45	1.19
	1.9	0.799	0.012	1.292	1.16	0.190	9.85	1.10
	2.2	0.800	0.007	1.259	1.18	0.193	9.75	1.14
	6.2	0.769	0.000	1.185	1.22	0.231	8.98	1.08
	10.3	0.748	0.004	1.162	1.30	0.248	9.30	1.11
	10.8	0.763	0.007	1.224	1.29	0.230	9.22	1.23
	12.6	0.773	0.004	1.181	1.34	0.222	9.07	1.12
	14.7	0.742	0.005	1.095	1.49	0.253	8.49	1.12
	14.8	0.733	0.004	1.090	1.51	0.263	8.37	1.10
	17.5	0.719	0.003	0.919	1.85	0.279	6.73	1.18
	19.5	0.703	0.005	0.892	2.08	0.292	6.50	1.14
	20.7	0.677	0.008	0.852	2.22	0.315	6.26	1.16
	21.7	0.674	0.002	0.847	2.27	0.324	5.61	1.11
	24.3	0.672	0.009	0.819	2.41	0.319	5.05	1.13
	27.1	0.669	0.013	0.749	2.63	0.318	5.73	1.14
	28.9	0.668	0.011	0.783	2.67	0.321	3.55	1.09
	36.0	0.633	0.012	0.792	2.75	0.355	3.70	1.05

*The decay was fitted with *globmis90obg-2*.

Table S3. Parameters retrieved from the global FBM analysisof the monomer decays with the program *globmis90sbg-2* for Py(5.8)-NAF(20) and Py(5.8)-NAF(8) at different NAF(56) concentrations in DMSO with Equations S1 and S2.

Sample	*wt* %	f_{Mdiff}	f_{Mfree}	k_{blob} ($\times 10^7 s^{-1}$)	<*n*>	f_{k2}	$k_e[blob]$ ($\times 10^6 s$-1)	$\chi 2$
Py(5.7)-NAF(20) k_2=1.8×10⁸ s⁻¹	0.0	0.815	0.014	1.204	1.04	0.172	9.66	1.14
	0.1	0.834	0.006	1.237	1.05	0.160	9.35	1.16
	1.2	0.814	0.016	1.315	1.02	0.170	10.06	1.10
	5.0	0.808	0.013	1.282	1.03	0.179	9.99	1.06
	8.6	0.780	0.014	1.130	1.14	0.205	9.65	1.14
	11.9	0.779	0.014	1.086	1.22	0.207	9.13	1.16
	16.8	0.752	0.010	0.959	1.38	0.238	7.46	1.18
	20.2	0.741	0.019	0.974	1.44	0.240	7.45	1.00
	22.4	0.727	0.016	0.950	1.53	0.257	6.30	1.17
	23.9	0.697	0.034	1.006	1.57	0.269	6.66	1.25
	27.8	0.687	0.031	0.975	1.66	0.282	5.49	1.16
	29.2	0.704	0.011	0.945	1.61	0.285	4.44	1.15
	32.2	0.687	0.020	1.038	1.58	0.282	5.06	1.16
	33.5	0.688	0.020	0.983	1.66	0.292	3.90	1.18
	37.2	0.704	0.027	0.971	1.66	0.268	5.66	1.28
*Py(*5.8)-NAF(8) k_2=2.0×10⁸ s⁻¹	0.1	0.802	0.041	1.335	1.01	0.156	9.33	1.29
	0.9	0.801	0.030	1.441	0.96	0.169	9.57	1.21
	4.8	0.780	0.032	1.353	0.97	0.188	9.49	1.17
	9.5	0.752	0.031	1.289	1.03	0.217	9.19	1.18
	12.9	0.735	0.026	1.131	1.15	0.239	8.32	1.22
	16.6	0.735	0.034	1.168	1.19	0.231	9.00	1.10
	19.9	0.735	0.033	1.177	1.23	0.232	9.18	1.14
	22.5	0.702	0.036	1.070	1.29	0.262	7.79	1.12
	26.2	0.688	0.073	1.173	1.19	0.239	8.54	1.29
	24.2	0.692	0.050	1.189	1.22	0.258	7.77	1.10
	31.1	0.682	0.042	1.090	1.26	0.276	6.46	1.22
	33.6	0.687	0.039	1.018	1.36	0.274	6.08	1.25
	34.8	0.665	0.042	1.069	1.32	0.292	5.47	1.17
	37.2	0.596	0.058	0.934	1.45	0.346	5.69	1.22

Table S4. Parameters retrieved from the global FBM analysis with the program *globmis90sbg-2* for the excimer decays of Py(4.1)-Amylopectin and Py(6.6)-NAF(56) at different NAF(56) concentrations in DMSO with Equations S1 and S2. τ_{ES} was fixed at 3.5 ns in the analysis.

Samples	*wt* %	τ_{E0} (ns)	τ_{EL} (ns)	$f_{Ediff, ES}$	$f_{Ediff, EL}$	f_{Ek2}	$f_{EE0, ES}$	$f_{EE0, EL}$	f_{ES}	$\chi 2$
Py(4.1)-Amylopectin (220) k_2=2.1×10^8 s^{-1}	0.0	50.4		0.662		0.088	0.093		0.157	1.16
	0.1	49.3		0.632		0.079	0.105		0.184	1.11
	1.2	48.5		0.625		0.089	0.090		0.196	1.17
	4.9	48.5		0.604		0.092	0.087		0.218	1.13
	8.6	47.8		0.576		0.114	0.060		0.250	1.05
	11.8	47.5		0.553		0.123	0.051		0.273	1.07
	16.6	47.5		0.514		0.129	0.048		0.309	1.12
	19.9	48.3		0.499		0.139	0.034		0.327	1.17
	22.5	48.2		0.458		0.147	0.036		0.360	1.10
	26.0	51.7		0.450		0.127	0.072		0.351	1.21
	27.8	50.9		0.416		0.155	0.030		0.399	1.11
	30.8	51.9		0.403		0.145	0.058		0.394	1.22
	32.8	51.5		0.371		0.168	0.017		0.444	1.23
	34.5	52.7		0.383		0.140	0.083		0.394	1.09
	36.6	52.1		0.353		0.154	0.050		0.443	1.09
	*41.3	50.6	100.0	0.045	0.007	0.151	0.010	0.309	0.477	1.12
*Py(*6.6)-NAF(56) k_2=2.0×10^8 s^{-1}	0.0	46.5		0.654		0.133	0.111		0.101	1.17
	0.1	46.3		0.648		0.139	0.105		0.109	1.21
	0.5	46.5		0.645		0.134	0.117		0.104	1.25
	1.3	46.3		0.634		0.148	0.092		0.126	1.19
	1.9	46.0		0.629		0.149	0.095		0.127	1.10
	2.2	46.3		0.626		0.151	0.093		0.130	1.14
	6.2	46.2		0.582		0.175	0.055		0.189	1.08
	10.3	46.8		0.569		0.182	0.048		0.220	1.11
	10.8	47.0		0.554		0.167	0.067		0.213	1.23
	12.6	47.1		0.553		0.159	0.081		0.208	1.12
	14.7	47.3		0.521		0.177	0.058		0.243	1.12
	14.8	47.4		0.511		0.183	0.050		0.256	1.10
	17.5	47.2		0.482		0.187	0.052		0.279	1.18
	19.5	48.0		0.459		0.191	0.061		0.289	1.14
	20.7	47.7		0.436		0.203	0.045		0.316	1.16
	21.7	47.9		0.425		0.204	0.044		0.327	1.11
	24.3	48.8		0.409		0.194	0.059		0.338	1.13
	27.1	49.5		0.413		0.196	0.074		0.316	1.14
	28.9	50.1		0.384		0.184	0.070		0.362	1.09
	36.0	51.1		0.327		0.184	0.078		0.411	1.05

*The ddecay was fitted with *globmis90obg-2*.

Table S5. Parameters retrieved from the global FBM analysis with the program *globmis90sbg-2* for the excimer decays of Py(5.7)-NAF(20) and Py(5.8)-NAF(8) at different NAF(56) concentrations in DMSO with Equations S1 and S2. τ_{ES} was fixed at 3.5 ns in the analysis.

Samples	*wt* %	τ_{E0} (ns)	f_{Ediff}	f_{Ek2}	f_{EE0}	f_{ES}	$\chi 2$
Py(5.7)-NAF(20)<br><br>k_2=1.8×10^8 s^{-1}	0.0	45.3	0.659	0.139	0.092	0.110	1.14
	0.1	45.5	0.663	0.127	0.114	0.096	1.16
	1.2	45.9	0.641	0.134	0.104	0.120	1.10
	5.0	45.8	0.623	0.138	0.094	0.145	1.06
	8.6	45.1	0.591	0.156	0.066	0.187	1.14
	11.9	45.8	0.572	0.153	0.072	0.202	1.16
	16.8	46.3	0.535	0.169	0.048	0.247	1.18
	20.2	47.0	0.512	0.166	0.054	0.268	1.00
	22.4	47.7	0.484	0.172	0.047	0.297	1.17
	23.9	48.4	0.452	0.174	0.047	0.327	1.25
	27.8	49.4	0.433	0.177	0.050	0.340	1.16
	29.2	49.3	0.421	0.171	0.046	0.363	1.15
	32.2	60.7	0.400	0.162	0.062	0.376	1.16
	33.5	50.8	0.386	0.164	0.061	0.389	1.18
	37.2	49.0	0.436	0.166	0.061	0.338	1.28
Py(5.8)-NAF(8)<br><br>k_2=2.0×10^8 s^{-1}	0.1	47.3	0.573	0.112	0.118	0.197	1.29
	0.9	47.6	0.562	0.119	0.109	0.210	1.21
	4.8	46.6	0.542	0.130	0.084	0.243	1.17
	9.5	46.7	0.504	0.145	0.065	0.285	1.18
	12.9	46.4	0.481	0.156	0.054	0.309	1.22
	16.6	46.1	0.467	0.147	0.061	0.327	1.10
	19.9	46.3	0.457	0.144	0.066	0.333	1.14
	22.5	47.4	0.432	0.161	0.042	0.365	1.12
	26.2	51.8	0.434	0.151	0.081	0.340	1.29
	24.2	49.3	0.404	0.151	0.047	0.398	1.10
	31.1	50.0	0.384	0.155	0.036	0.424	1.22
	33.6	49.8	0.368	0.147	0.043	0.442	1.25
	34.8	51.5	0.358	0.157	0.040	0.444	1.17
	37.2	52.4	0.310	0.180	0.007	0.502	1.22

Table S6. Molar fractions of the pyrene species of Py(4.1)-Amylopectin(220) and Py(6.6)-NAF(56) at different NAF(56) concentrations in DMSO calculated from f_{Mfree}, f_{Mdiff}, f_{Ediff}, f_{k2} and f_{E0}.

Samples	*wt* %	f_{diff}	f_{k2}	f_{free}	f_{E0}
Py(4.1)-Amylopectin (220) k_2=2.1×10^8 s^{-1}	0.0	0.753	0.100	0.042	0.105
	0.1	0.733	0.091	0.054	0.122
	1.2	0.747	0.107	0.039	0.107
	4.9	0.737	0.112	0.045	0.106
	8.6	0.740	0.146	0.037	0.077
	11.8	0.731	0.163	0.038	0.068
	16.6	0.719	0.180	0.034	0.067
	19.9	0.714	0.199	0.038	0.049
	22.5	0.691	0.221	0.034	0.054
	26.0	0.649	0.183	0.065	0.103
	27.8	0.653	0.243	0.057	0.047
	30.8	0.620	0.223	0.068	0.089
	32.8	0.628	0.285	0.057	0.029
	34.5	0.587	0.215	0.070	0.128
	36.6	0.592	0.258	0.065	0.084
	41.3	0.551	0.261	0.097	0.092
*Py(*6.6)-NAF(56) k_2=2.0×10^8 s^{-1}	0.0	0.724	0.147	0.005	0.123
	0.1	0.725	0.155	0.003	0.117
	0.5	0.711	0.148	0.012	0.129
	1.3	0.721	0.169	0.006	0.104
	1.9	0.713	0.169	0.010	0.108
	2.2	0.715	0.172	0.006	0.107
	6.2	0.717	0.215	0.000	0.067
	10.3	0.703	0.233	0.004	0.060
	10.8	0.699	0.210	0.006	0.084
	12.6	0.695	0.200	0.004	0.101
	14.7	0.685	0.233	0.005	0.077
	14.8	0.684	0.245	0.004	0.068
	17.5	0.667	0.259	0.003	0.072
	19.5	0.643	0.267	0.005	0.086
	20.7	0.633	0.295	0.008	0.065
	21.7	0.630	0.302	0.002	0.066
	24.3	0.613	0.290	0.008	0.089
	27.1	0.597	0.284	0.012	0.108
	28.9	0.595	0.286	0.010	0.109
	36.0	0.550	0.308	0.010	0.132

Table S7. Molar fractions of the pyrene species of Py(5.7)-NAF(20) and Py(5.8)-NAF(8) at different NAF(56) concentrations in DMSO calculated from f_{Mfree}, f_{Mdiff}, f_{Ediff}, f_{k2} and f_{E0}.

Sample	*wt* %	f_{diff}	f_{k2}	f_{free}	f_{E0}
Py(5.7)-NAF(20)<br>k_2=1.8×10^8 s^{-1}	0.0	0.732	0.154	0.012	0.102
	0.1	0.730	0.140	0.005	0.125
	1.2	0.719	0.150	0.014	0.117
	5.0	0.720	0.160	0.012	0.108
	8.6	0.717	0.189	0.013	0.080
	11.9	0.709	0.189	0.013	0.090
	16.8	0.704	0.223	0.009	0.064
	20.2	0.687	0.222	0.018	0.072
	22.4	0.679	0.241	0.015	0.065
	23.9	0.650	0.251	0.032	0.067
	27.8	0.637	0.261	0.029	0.074
	29.2	0.653	0.265	0.010	0.071
	32.2	0.626	0.257	0.019	0.098
	33.5	0.620	0.264	0.018	0.098
	37.2	0.641	0.244	0.025	0.089
Py(5.7)-NAF(8)<br>k_2=2.0×10^8 s^{-1}	0.1	0.689	0.134	0.036	0.141
	0.9	0.693	0.146	0.026	0.135
	4.8	0.696	0.167	0.028	0.108
	9.5	0.685	0.197	0.029	0.088
	12.9	0.679	0.220	0.024	0.077
	16.6	0.670	0.211	0.031	0.088
	19.9	0.665	0.210	0.030	0.096
	22.5	0.657	0.245	0.034	0.063
	26.2	0.610	0.212	0.065	0.114
	24.2	0.640	0.238	0.047	0.075
	31.1	0.641	0.259	0.039	0.060
	33.6	0.637	0.254	0.036	0.074
	34.8	0.619	0.272	0.039	0.070
	37.2	0.588	0.341	0.057	0.013

Table S8. Parameters retrieved from the global FBM analysis with the program *globmis90sbg-2* for the monomer decays of Py(6.6)-NAF(56) at different Py(6.6)-NAF(56) concentrations in DMSO with Equations S1 and S2.

Sample	*wt* %	τ_M (ns)	f_{Mdiff}	f_{Mfree}	k_{blob} ($\times 10^7 s^{-1}$)	<*n*>	f_{k2}	$k_e[blob]$ ($\times 10^6 s$-1)	$\chi 2$
Py(6.6)-NAF(56) k_2=2.0×10⁸ s⁻¹	0.1	100.2	0.824	0.020	1.560	1.07	0.157	11.00	1.17
	0.7	100.1	0.812	0.004	1.384	1.06	0.184	9.91	1.20
	3.0	100.0	0.769	0.002	1.480	1.04	0.229	7.40	1.28
	4.2	100.0	0.774	0.007	1.419	1.09	0.219	14.19	1.14
	6.1	100.3	0.777	0.002	1.488	1.12	0.221	8.84	1.17
	6.5	100.4	0.783	0.006	1.421	1.08	0.211	7.81	1.02
	11.3	102.1	0.622	0.000	1.015	1.65	0.378	6.41	1.15
	11.3	102.2	0.725	0.000	1.047	1.52	0.275	5.97	1.28
	14.8	104.0	0.751	0.000	1.078	1.95	0.249	8.09	1.18
	21.1	108.6	0.768	0.001	0.677	2.76	0.231	7.99	1.15
	*36.1	121.9	0.592	0.000	0.876	2.86	0.408	5.30	1.14

*The decay was fitted with *globmis90obg-2*.

Table S8. Parameters retrieved from the global FBM analysis with the program *globmis90sbg-2* for the excimer decays of Py(6.6)-NAF(56) at different Py(6.6)-NAF(56) concentrations in DMSO with Equations S1 and S2. τ_{ES} was fixed at 3.5 ns in the analysis.

Sample	*wt* %	τ_{E0} (ns)	τ_{EL} (ns)	$f_{Ediff, ES}$	$f_{Ediff, EL}$	f_{Ek2}	$f_{EE0, ES}$	$f_{EE0, EL}$	f_{ES}	$\chi 2$
*Py(*6.6)-NAF(56) k_2=2.0 ×10⁸ s⁻¹	0.1	47.6		0.667		0.127	0.152		0.055	1.17
	0.7	48.2		0.651		0.147	0.135		0.067	1.20
	3.0	53.0		0.589		0.175	0.138		0.099	1.28
	4.2	51.7		0.592		0.168	0.136		0.104	1.14
	6.1	51.4		0.585		0.167	0.133		0.115	1.17
	6.5	51.4		0.624		0.168	0.122		0.087	1.02
	11.3	51.1		0.470		0.286	0.007		0.236	1.15
	11.3	53.6		0.560		0.212	0.128		0.099	1.28
	14.8	49.9		0.515		0.171	0.217		0.096	1.18
	21.1	48.7		0.531		0.160	0.309		0.000	1.26
	*36.1	46.5	69.8	0.247	0.173	0.169	0.079	0.166	0.166	1.14

*The decay was fitted with *globmis90obg-2*.

Table S9. Molar fractions of the pyrene species of Py(6.6)-NAF(20) at different Py(6.6)-NAF(56) concentrations in DMSO calculated from f_{Mfree}, f_{Mdiff}, f_{Ediff}, f_{k2} and f_{E0}.

Sample	*wt* %	f_{diff}	f_{k2}	f_{free}	f_{E0}
Py(6.6)-NAF(56) k_2=2.0×10^8 s^{-1}	0.1	0.694	0.132	0.016	0.158
	0.7	0.695	0.157	0.004	0.144
	3.0	0.652	0.194	0.002	0.153
	4.2	0.657	0.186	0.006	0.151
	6.1	0.660	0.188	0.002	0.150
	6.5	0.679	0.183	0.005	0.133
	11.3	0.616	0.375	0.000	0.009
	11.3	0.622	0.236	0.000	0.142
	14.8	0.570	0.189	0.000	0.240
	21.1	0.530	0.160	0.001	0.309
	36.1	0.294	0.202	0.000	0.504

F] *Number of overlapping carbons and ΔN_{blob} values retrieved from MMOs*

Table S10. Number of overlapping carbons between the reference pyrene attached on Helix #0 with an angle $\varphi = 30^o$ and the secondary pyrene attached on Helix 2 at $\theta = 60^o$ for $d_{h\text{-}h}$ values ranging from 3.4 nm to 1.6 nm with the total number of AGUs (ΔN_{blob}) allowing a good pyrene overlap with 7 or more carbon atoms.

$d_{h\text{-}h}$, nm / Position	1.6 nm	1.8 nm	2.4 nm	2.5 nm	2.6 nm	2.7 nm	2.8 nm	2.9 nm	3.0 nm	3.1 nm	3.2 nm	3.3 nm	3.4 nm
-18	0	0	0	0	0	0	0	0	0	0	0	0	0
-17	0	0	0	0	0	0	0	0	0	0	0	0	0
-16	0	0	0	0	0	0	0	0	0	0	0	0	0
-15	0	0	0	0	0	0	0	0	0	0	0	0	0
-14	0	0	0	0	0	0	0	0	0	0	0	0	0
-13	0	0	0	0	0	0	0	0	0	0	0	0	0
-12	≥ 7	7	7	0	6	5	5	0	0	0	0	0	0
-11	≥ 7	≥ 7	≥ 7	≥ 7	≥ 7	≥ 7	8	≥ 7	8	6	0	0	0
-10	≥ 7	≥ 7	≥ 7	≥ 7	8	7	6	0	0	0	0	0	0
-9	0	0	0	0	0	0	0	0	0	0	0	0	0
-8	0	0	0	0	0	0	0	0	0	0	0	0	0
-7	0	0	0	0	0	0	0	0	0	0	0	0	0
-6	0	0	0	0	0	0	0	0	0	0	0	0	0
-5	≥ 7	≥ 7	≥ 7	≥ 7	≥ 7	≥ 7	7	≥ 7	7	0	0	0	0
-4	≥ 7	≥ 7	≥ 7	≥ 7	≥ 7	≥ 7	8	≥ 7	≥ 7	8	7	5	0
-3	≥ 7	≥ 7	≥ 7	≥ 7	≥ 7	≥ 7	8	≥ 7	≥ 7	7	7	6	0
-2	≥ 7	8	8	8	6	4	4	0	0	0	0	0	0
-1	0	0	0	0	0	0	0	0	0	0	0	0	0
0	0	0	0	0	0	0	0	0	0	0	0	0	0
+1	0	0	0	0	0	0	0	0	0	0	0	0	0
+2	≥ 7	≥ 7	≥ 7	≥ 7	≥ 7	≥ 7	7	6	5	0	0	0	0
+3	≥ 7	≥ 7	≥ 7	≥ 7	≥ 7	≥ 7	8	≥ 7	≥ 7	7	7	0	0
+4	≥ 7	≥ 7	≥ 7	≥ 7	≥ 7	≥ 7	10	8	5	6	0	0	0
+5	≥ 7	≥ 7	≥ 7	≥ 7	7	4	4	0	0	0	0	0	0
+6	0	0	0	0	0	0	0	0	0	0	0	0	0
+7	0	0	0	0	0	0	0	0	0	0	0	0	0
+8	0	0	0	0	0	0	0	0	0	0	0	0	0
+9	≥ 7	≥ 7	≥ 7	≥ 7	7	5	5	0	0	0	0	0	0
+10	≥ 7	≥ 7	≥ 7	≥ 7	≥ 7	≥ 7	8	≥ 7	≥ 7	7	0	8	0
+11	≥ 7	7	7	7	0	0	0	0	0	0	0	0	0
+12	0	0	0	0	0	0	0	0	0	0	0	0	0
+13	0	0	0	0	0	0	0	0	0	0	0	0	0
+14	0	0	0	0	0	0	0	0	0	0	0	0	0
+15	0	0	0	0	0	0	0	0	0	0	0	0	0
+16	0	0	0	0	0	0	0	0	0	0	0	0	0
+17	0	0	0	0	0	0	0	0	0	0	0	0	0

$N_{blob,\,30}$	14	14	14	13	11	9	8	7	6	4	3	1	0

* The AGUs with ≥ were counted toward N_{blob} without conducting the MMO, because the pyrenyls attached at the same positions had been shown to generate a good overlap even at a larger distance.

Table S11. Number of overlapping carbons between the reference pyrene attached on Helix #0 with an angle $\varphi = 30^{o}$ and the secondary pyrene attached on Helix 3 at θ = 120° for $d_{h\text{-}h}$ values ranging from 3.4 nm to 1.6 nm with the total number of AGUs (ΔN_{blob}) allowing a good pyrene overlap with 7 or more carbon atoms.

$d_{h\text{-}h}$, nm / Position	1.6 nm	1.8 nm	2.5 nm	2.6 nm	2.7 nm	2.8 nm	2.9 nm	3.0 nm	3.1 nm	3.2 nm	3.3 nm	3.4 nm
-15	0	0	0	0	0	0	0	0	0	0	0	0
-14	0	0	0	0	0	0	0	0	0	0	0	0
-13	0	0	0	0	0	0	0	0	0	0	0	0
-12	0	0	0	0	0	0	0	0	0	0	0	0
-11	0	0	0	0	0	0	0	0	0	0	0	0
-10	0	0	0	0	0	0	0	0	0	0	0	0
-9	≥ 7	≥ 7	≥ 7	≥ 7	≥ 7	7	8	7	4	0	0	0
-8	≥ 7	8	7	5	4	4	0	0	0	0	0	0
-7	0	0	0	0	0	0	0	0	0	0	0	0
-6	0	0	0	0	0	0	0	0	0	0	0	0
-5	0	0	0	0	0	0	0	0	0	0	0	0
-4	0	0	0	0	0	0	0	0	0	0	0	0
-3	≥ 7	≥ 7	≥ 7	≥ 7	≥ 7	9	≥ 7	7	5	0	0	0
-2	≥ 7	≥ 7	≥ 7	≥ 7	≥ 7	8	≥ 7	≥ 7	7	6	0	0
-1	≥ 7	≥ 7	≥ 7	≥ 7	≥ 7	7	7	6	5	0	0	0
0	0	0	0	0	0	0	0	0	0	0	0	0
+1	0	0	0	0	0	0	0	0	0	0	0	0
+2	0	0	0	0	0	0	0	0	0	0	0	0
+3	0	0	0	0	0	0	0	0	0	0	0	0
+4	≥ 7	≥ 7	≥ 7	≥ 7	≥ 7	7	≥ 7	7	4	0	0	0
+5	≥ 7	≥ 7	≥ 7	≥ 7	≥ 7	8	≥ 7	≥ 7	7	8	0	0
+6	≥ 7	≥ 7	≥ 7	7	7	3	0	0	0	0	0	0
+7	0	0	0	0	0	0	0	0	0	0	0	0
+8	0	0	0	0	0	0	0	0	0	0	0	0
+9	0	0	0	0	0	0	0	0	0	0	0	0
+10	0	0	0	0	0	0	0	0	0	0	0	0
+11	≥ 7	≥ 7	≥ 7	≥ 7	≥ 7	8	5	4	0	0	0	0
+12	≥ 7	≥ 7	≥ 7	≥ 7	≥ 7	7	≥ 7	7	4	0	0	0
+13	0	0	0	0	0	0	0	0	0	0	0	0
+14	0	0	0	0	0	0	0	0	0	0	0	0

+15	0	0	0	0	0	0	0	0	0	0	0	0
+16	0	0	0	0	0	0	0	0	0	0	0	0
+17	0	0	0	0	0	0	0	0	0	0	0	0
+18	0	0	0	0	0	0	0	0	0	0	0	0
$N_{\text{blob, 90}}$	10	10	10	9	9	8	7	6	2	1	0	0

* The AGUs with ≥ were counted toward N_{blob} without conducting an MMO because the pyrenyl labels at the same positions had been shown to generate a good overalp even at a larger distance.
